# Community and clinical resistomes and mobilomes: a comparative genomic snapshot of *Escherichia coli* and *Klebsiella pneumoniae*

**DOI:** 10.1038/s41598-026-64511-x

**Published:** 2026-07-28

**Authors:** Khanyisa Mahonisi, Joshua Mbanga, Arshad Ismail, Sabiha Y. Essack

**Affiliations:** 1 Antimicrobial Research Unit, College of Health Sciences, University of KwaZulu-Natal, Durban, South Africa; 2 Department of Applied Biology and Biochemistry, National University of Science and Technology, AC939, Ascot, Bulawayo, Zimbabwe; 3 Sequencing Core Facility, National Institute for Communicable Diseases, National Health Laboratory Service, Johannesburg, South Africa; 4 Department of Biochemistry and Microbiology, Faculty of Science, Engineering and Agriculture, University of Venda, Thohoyandou, South Africa; 5 School of Pharmacy, University of Jordan, Amman, Jordan

**Keywords:** Community resistome, Clinical resistome, Carriage, *Escherichia coli*, *Klebsiella pneumoniae*, Antimicrobial resistance, Genetics, Microbiology

## Abstract

*Escherichia coli* and *Klebsiella pneumoniae* are major contributors to antimicrobial resistance (AMR)-related mortality globally. The resistomes, mobilomes, clonalities, and phylogenies of 32 *E. coli* (15 clinical, 17 community) and 14 *K. pneumoniae* (6 clinical, 8 community) isolates from KwaZulu-Natal were compared to ascertain similarities and differences between healthcare-related pathogens and community commensals using whole genome sequencing and bioinformatic pipelines. Resistome analysis revealed that *bla*_CTX−M−15_ predominated in clinical isolates of both *E. coli* and *K. pneumoniae*, whereas *bla*_TEM−1B_ was more common in community *E. coli*. Two clinical *E. coli* harboured *bla*_OXA−181_, and one *K. pneumoniae* carried *bla*_OXA−181_ and *bla*_NDM−5_; none were detected in community isolates. IncF plasmid replicons were most frequent, and gene cassettes within class 1 integrons, insertion sequences, and transposons contributed to resistome mobilization across both settings. A broad range of sequence types (STs) were detected across both settings, with shared STs limited to ST131, ST10, and ST1193 in *E. coli*, and ST17 in *K. pneumoniae*. Phylogeny showed that clinical *E. coli* clustered with some community isolates, while *K. pneumoniae* showed limited clustering. The overlap of resistomes, mobilomes, STs and phylogenies suggests genomic relatedness between isolates from both settings, highlighting the importance of AMR surveillance in community settings.

**Supplementary Information:**

The online version contains supplementary material available at https://doi.org/10.1038/s41598-026-64511-x.

## Introduction

Antimicrobial resistance (AMR) remains a persistent global public health threat, undermining the effective management of infectious diseases and imposing considerable constraints on medical progress^1,2^. Determining the true burden of AMR remains a significant challenge, particularly in settings where surveillance systems are weak and data availability is limited^3^. Sub-Saharan Africa is projected to bear a high burden of AMR, with an estimated 6.63 million deaths (95% uncertainty interval 5.00-8.66) directly attributable to AMR expected to occur in the region from 2025 to 2050^4^. *Escherichia coli* and *Klebsiella pneumoniae* are opportunistic Enterobacterales pathogens^5^. In 2019, they were among the six leading pathogens responsible for AMR-related deaths globally, in which *E. coli* was the leading cause of death^3^.

Human-to-human interface plays a significant role in the transmission and acquisition of AMR in both healthcare and community settings^6^. Other studies have reported a variety of antibiotic resistance genes (ARGs) and mobile genetic elements (MGEs) in *E. coli* and *K. pneumoniae* clinical isolates in South Africa conferring resistance to a wide range of antibiotics, such as aminoglycosides, β-lactams, polymyxins, and fluoroquinolones, among others^7–14^. The number of individuals colonized by antibiotic-resistant bacteria without symptoms far exceeds those with active infections, suggesting that AMR surveillance in healthy community dwellers could serve as a valuable early warning system for detecting emerging resistance mechanisms that contribute to the broader AMR burden^15^. This is further supported by a longitudinal study by Hilty et al.^16^, showed that the transmission rates of extended-spectrum β-lactamase-producing (ESBL) *E. coli* and *K. pneumoniae* were significantly higher within households than in the hospital setting.

The acquisition of ARGs across bacterial species is largely attributable to MGEs^17^, with most ARGs located on MGEs rather than on the bacterial chromosome^18^. Symptomatic patients and asymptomatic carriers are both reservoirs of AMR dissemination^18^. Therefore, delineating the presence of ARGs along with their association to MGEs is essential for understanding the transmission dynamics of AMR in both clinical and community settings. This study compares the prevalence of AMR, the distribution of ARGs and MGEs, as well as the clonal and phylogenetic relationships among *E. coli* and *K. pneumoniae* isolates collected from patients at a regional hospital and community dwellers residing in proximity to the hospital in KwaZulu-Natal using whole genome sequencing (WGS) and bioinformatics analysis.

## Methods

### Ethical approval and participant consent

This study was ethically approved by the Biomedical Research Ethics Committee of the University of KwaZulu-Natal (Reference No. BREC 00003640 − 2021) and the Provincial Health Research Ethics Committee of the KwaZulu-Natal Department of Health (Reference No. KZ 202203 023). All research was performed in accordance with relevant guidelines/regulations including the Declaration of Helsinki. Written informed consent was obtained from all community participants prior to enrolment.

### Study site and sample collection

This study is nested within a broader, ongoing longitudinal AMR surveillance study that was conducted among 300 participants residing in informal settlements over a 12-month period (March 2023 - February 2024). The study sites were informal settlements in Ward 22 in the uMgungundlovu District, which is one of the 11 districts in KwaZulu-Natal^19^. *E. coli* and *K. pneumoniae* were selectively isolated from stool samples of healthy community dwellers, which were collected monthly during the same period. Stool samples were self-collected by participants following standardized instructions and placed into sterile containers provided by trained study personnel. Participants were considered healthy community dwellers based on self-reported wellness and no formal clinical symptom screening was performed. Additionally, samples were transported the same day to a public clinical laboratory under cold-chain conditions (4 °C) and processed within 24 h. Sterile techniques were maintained throughout collection, handling, and transport to minimise contamination.

The National Health Laboratory Services (NHLS) processed and provided the clinical samples that were collected from inpatients and outpatients at a regional hospital near the informal settlement. The hospital departments from which the samples were obtained included the intensive care unit (ICU), outpatient department (OPD), emergency department, gynaecology OPD, orthopaedic, paediatric, surgical, and other medical wards. The clinical specimen types included urine, blood cultures, sputum, tissue, pus, and other sterile fluids and sites. This study constitutes clinical and community isolates collected in December 2023, with the total sample size of 35 clinical isolates (22 *E. coli* and 13 *K. pneumoniae*), which was matched with a proportionate number of randomly-selected isolates from the community (44 *E. coli* and 26 *K. pneumoniae*). Furthermore, from this pool, 61 isolates were selected for WGS based on phenotypic resistance to at least one antibiotic class. This selection criterion was applied consistently across both clinical and community isolates. Consequently, 29 clinical (20 *E. coli* and 9 *K. pneumoniae*) and 32 community (22 *E. coli* and 10 *K. pneumoniae*) were subjected to WGS. Following WGS, 15 isolates were excluded from this study due to cross-contamination with other bacterial species. The 15 isolates consisted of 8 clinical (5 *E. coli* and 3 *K. pneumoniae*) and 7 community (5 *E. coli* and 2 *K. pneumoniae*) isolates.

### Bacterial identification and antibiotic susceptibility profiling

*E. coli* isolates were cultured on CHROMagar *E. coli*/Coliforms (ECC) (Paris, France) plates, incubated for 24 h at 37 °C. *K. pneumoniae* were cultured on Simmons’ Citrate Agar (Merck, Darmstadt, Germany) plates that were supplemented with Inositol (SCAI), adjusted to pH 7.2, and incubated at 37 °C for 48 h. Antibiotic susceptibility was assessed using the AST-N256 VITEK 2 system card (BioMérieux, Durham, North Carolina, USA) on the VITEK 2 automated system (BioMérieux, Marcy-l’Étoile, France). The following antibiotic panel was used: amikacin (AMK), amoxicillin–clavulanic acid (AMC), ampicillin/amoxicillin (AMP/AMX), cefepime (CPM), cefotaxime/ceftriaxone (CTX/CRO), ceftazidime (CAZ), cefuroxime (CXM), ciprofloxacin (CIP), ertapenem (ERT), gentamicin (GEN), imipenem (IMP), meropenem (MEM), piperacillin-tazobactam (TZP), tigecycline (TGC), and trimethoprim-sulfamethoxazole/co-trimoxazole (SXT). In addition, cefoxitin (FOX) and nitrofurantoin (NIT) were included in the antibiotic panel for clinical isolates, while doripenem (DOR) and tobramycin (TOB) were included for community isolates. Results were interpreted as either susceptible (S), intermediate (I), or resistant (R) according to the guidelines of the Clinical and Laboratory Standards Institute^20^. Community isolates were screened for ESBL production using CHROMID ESBL agar (BioMérieux, Marcy-l’Étoile, France) plates, incubated at 37 °C for 24 h. Clinical isolates were screened at the NHLS using the VITEK 2 automated system (BioMérieux, Marcy-l’Étoile, France), which applies the Advanced Expert System (AES) to detect potential ESBL-producers based on cephalosporin minimum inhibitory concentration patterns.

### WGS and annotation

Genomic DNA was extracted using the GenElute Bacterial Genomic DNA kit (Sigma-Aldrich, St. Louis, MO, USA) according to the manufacturer’s instructions. The NanoDrop Lite Plus Spectrophotometer (Thermo Fisher Scientific, Waltham, Massachusetts, USA) was used to assess the quality and quantity of the gDNA. WGS was performed at the National Institute of Communicable Diseases (NICD). Paired-end libraries (2 × 300 bp) were prepared and multiplexed using the Illumina DNA Prep Kit (Illumina, San Diego, CA, USA), and sequencing was conducted on the Illumina NextSeq 2000 System. Raw reads were trimmed using Trim-Galore v0.6.10 (https://github.com/FelixKrueger/TrimGalore), and *de novo* assembly was performed using SKESA v2.3.0 (https://github.com/ncbi/SKESA). The assembled genomes were submitted to the National Center for Biotechnology Information (NCBI) GenBank database under the BioProject PRJNA1183563, with individual accession numbers provided in Supplementary Table S1.

### Genome, resistome, mobilome and clonal analysis

RAST SEEDVIEWER (https://rast.nmpdr.org/seedviewer.cgi) was used to determine the genome characteristics. Resistome (ARGs, chromosomal point mutations and efflux pumps) and mobilome (plasmids, insertion sequences, integrons, gene cassettes, and transposons) were characterized using bioinformatics tools. ARGs and chromosomal point mutations were identified using ResFinder^21,22^, at a 90% threshold ID and 60% minimum length. The Comprehensive Antibiotic Resistance Database (CARD) (https://card.mcmaster.ca/) was used to identify efflux pump genes^23^. Multilocus Sequence Typing (MLST) was performed using MLST 2.0 (https://cge.food.dtu.dk/services/MLST/). Plasmid replicons were detected using PlasmidFinder 2.1 (https://cge.food.dtu.dk/services/PlasmidFinder/) at a 95% threshold ID and 60% minimum coverage. The INTEGRALL database (http://integrall.bio.ua.pt/) was used to identify integrons and gene cassettes. Insertion sequences (ISs) were delineated using ISFinder (https://isfinder.biotoul.fr/). The NCBI Prokaryotic Genome Annotation Pipeline (PGAP) annotation files (https://www.ncbi.nlm.nih.gov/bioproject/?term=PRJNA1183563) were used to assess the synteny of ISs and transposons (Tns) with ARGs.

### Phylogenetic relationships

Publicly available South African clinical whole genome sequences of *E. coli* (*n* = 50, 2016–2018, 2020 and 2023) and *K. pneumoniae* (*n* = 131, 2019–2024) isolates curated on the Bacterial and Viral Bioinformatics Resource Center (BV-BRC) platform (https://www.bv-brc.org/), were retrieved and compared with genomes sequenced in this study to construct phylogenetic trees. The phylogenetic trees were constructed using Bacterial Genome Tree (https://www.bv-brc.org/app/PhylogeneticTree) on the BV-BRC platform. The phylogenetic trees were constructed using a maximum-likelihood codon-based approach based on conserved genes shared across the genomes. All trees were visualized, annotated, and edited using the Interactive Tree of Life (iTOL) online tool (https://itol.embl.de/)^24^.

### Statistical analysis

All analyses were performed in RStudio (version 4.5.1). Phenotypic antimicrobial susceptibility testing (AST) results were summarised as source-specific frequencies and percentages of susceptible, intermediate, and resistant isolates. For comparative analyses, only antibiotics (*n* = 15) common to both clinical and community isolates were included. Differences in phenotypic resistance and antimicrobial resistance gene (ARG) prevalence between sources were assessed using chi-square tests, with Fisher’s exact test applied where expected cell counts were small. False discovery rate (FDR) correction was applied to account for multiple comparisons. Overall phenotypic and resistome compositions were compared between sources using PERMANOVA based on Jaccard distances. Statistical analyses were exploratory, complementing the descriptive nature of the study, with significance set at *p* < 0.05.

## Results

### Prevalence of confirmed isolates and participant demographics

This study reports on 32 *E. coli* (*n* = 15 clinical; *n* = 17 community) and 14 *K. pneumoniae* (*n* = 6 clinical; *n* = 8 community) isolates that were confirmed by WGS. Clinical isolates were sourced from various hospital departments, including the medical ward (*n* = 5), paediatric ward (*n* = 1), emergency department (*n* = 4), gynaecology OPD (*n* = 3), surgical ward (*n* = 3), and other inpatient departments (*n* = 5). The age distribution of community participants (excluding two with unknown age) ranged from 22 to 65 years, with a mean age of 40.9. For clinical participants, age ranged from 0 to 92 years, with a mean age of 39.5 years. The demographic details of the participants and bacterial isolate characteristics are shown in Supplementary Table S1, while the clinical isolate source by hospital department is detailed within the main text.

### Antibiotic resistance profile of isolates

All *E. coli* and *K. pneumoniae* were susceptible to amikacin and ertapenem (Table 1). Among the 32 *E. coli* isolates, 31 (96.9%) exhibited resistance to at least one β-lactam antibiotic, including penicillins (AMP/AMX and AMC), 2nd to 4th generation cephalosporins (CXM, CTX/CRO, CAZ, and CPM), and cefoxitin (FOX). Resistance was also observed to non-β-lactam antibiotics, including aminoglycoside (GEN), β-lactamase inhibitor (TZP), fluoroquinolone (CIP), and trimethoprim-sulfamethoxazole (SXT). High resistance to SXT (86.7% clinical, 82.4% community) was observed, followed by CXM (73.3% clinical, 23.5% community), CIP (73.3% clinical, 17.6% community), and CTX/CRO (60% clinical, 23.5% community). Notably, CIP resistance differed significantly between clinical and community *E. coli* isolates (*p* = 0.00103), while no other antibiotics showed significant differences (*p* > 0.05) (Supplementary Table S5). One clinical isolate exhibited intermediate susceptibility to IMP and MEM, while one community isolate was resistant to MEM (Table 1).

Clinical *K. pneumoniae* isolates showed high resistance to CTX/CRO (83.3%), CXM (83.3%), CAZ (83.3%), SXT (83.3%), AMC (66.7%), TZP (66.7%), CIP (66.7%), GEN (66.7%), and FOX (50%). In contrast, community *K. pneumoniae* isolates showed narrower resistance profiles, with resistance to TGC (50%) being most prominent, and moderate resistance to TZP, CIP, and SXT (25%) was observed. One clinical isolate showed resistance to IMP and MEM, while one community isolate showed resistance to MEM (Table 1). Although clinical isolates showed higher resistance prevalence for several antibiotics, no significant differences were observed between clinical and community *K. pneumoniae* isolates (all *p* > 0.05) (Supplementary Table S5).

**Table 1:** Antimicrobial susceptibility of clinical and community *E. coli* and *K. pneumoniae* isolates using VITEK 2 AES.

Antibiotic panel by class	*E. coli* clinical (*n* = 15)			*E. coli* community (*n* = 17)			*K. pneumoniae* clinical (*n* = 6)			*K. pneumoniae* community (*n* = 8)		
	S	I	*R*	S	I	*R*	S	I	*R*	S	I	*R*
Aminoglycoside												
AMK	15 (100)	0 (0)	0 (0)	17 (100)	0 (0)	0 (0)	6 (100)	0 (0)	0 (0)	8 (100)	0 (0)	0 (0)
GEN	13 (86.7)	0 (0)	2 (13.3)	17 (100)	0 (0)	0 (0)	2 (33.3)	0 (0)	4 (66.7)	8 (100)	0 (0)	0 (0)
TOB	−	−	−	17 (100)	0 (0)	0 (0)	−	−	−	8 (100)	0 (0)	0 (0)
Penicillin & β-lactamase inhibitor												
AMP/AMX	0 (0)	0 (0)	15 (100)	3 (17.6)	0 (0)	14 (82.4)	0 (0)	0 (0)	6 (100)	0 (0)	0 (0)	8 (100)
AMC	11 (73.3)	2 (13.3)	2 (13.3)	12 (70.6)	1 (5.9)	4 (23.5)	2 (33.3)	0 (0)	4 (66.7)	6 (75.0)	1 (12.5)	1 (12.5)
TZP	13 (86.7)	0 (0)	2 (13.3)	12 (70.6)	0 (0)	5 (29.4)	2 (33.3)	0 (0)	4 (66.7)	6 (75.0)	0 (0)	2 (25.0)
Cephalosporin & cephamycin												
CPM	13 (86.7)	0 (0)	2 (13.3)	13 (76.5)	0 (0)	4 (23.5)	2 (33.3)	2 (33.3)	2 (33.3)	7(87.5)	0 (0)	1 (12.5)
CTX/CRO	6 (40.0)	0 (0)	9 (60.0)	13 (76.5)	0 (0)	4 (23.5)	1 (16.7)	0 (0)	5 (83.3)	7(87.5)	0 (0)	1 (12.5)
CXM	4 (26.7)	0 (0)	11 (73.3)	13 (76.5)	0 (0)	4 (23.5)	1 (16.7)	0 (0)	5 (83.3)	6 (75.0)	1 (12.5)	1 (12.5)
CAZ	10 (66.7)	1 (6.7)	4 (26.7)	13 (76.5)	0 (0)	4 (23.5)	1 (16.7)	0 (0)	5 (83.3)	7(87.5)	0 (0)	1 (12.5)
FOX	13 (86.7)	0 (0)	2 (13.3)	−	−	−	3 (50.0)	0 (0)	3 (50.0)	−	−	−
Fluoroquinolone												
CIP	3 (20.0)	1 (6.7)	11 (73.3)	14 (82.4)	0 (0)	3 (17.6)	2 (33.3)	0 (0)	4 (66.7)	5 (62.5)	1 (12.5)	2 (25.0)
Folate pathway inhibitor												
SXT	2 (13.3)	0 (0)	13 (86.7)	3 (17.6)	0 (0)	14 (82.4)	1 (16.7)	0 (0)	5 (83.3)	6 (75.0)	0 (0)	2 (25.0)
Carbapenems												
DOR	−	−	−	17 (100)	0 (0)	0 (0)	−	−	−	8 (100)	0 (0)	0 (0)
ERT	15 (100)	0 (0)	0 (0)	17 (100)	0 (0)	0 (0)	6 (100)	0 (0)	0 (0)	8 (100)	0 (0)	0 (0)
IMP	14 (93.3)	1 (6.7)	0 (0)	17 (100)	0 (0)	0 (0)	5 (83.3)	0 (0)	1 (16.7)	8 (100)	0 (0)	0 (0)
MEM	14 (93.3)	1 (6.7)	0 (0)	16 (94.1)	0 (0)	1 (5.9)	5 (83.3)	0 (0)	1 (16.7)	7(87.5)	0 (0)	1 (12.5)
Other												
TGC	15 (100)	0 (0)	0 (0)	17 (100)	0 (0)	0 (0)	6 (100)	0 (0)	0 (0)	4 (50.0)	0 (0)	4 (50.0)
NIT	15 (100)	0 (0)	0 (0)	−	−	−	2 (33.3)	2 (33.3)	2 (33.3)	−	−	−

Data is expressed as the number of isolates with the percentage in parentheses. The (-) symbol indicates that a particular antibiotic was not included in the respective antibiotic panel. Key: AMK= amikacin, GEN= gentamicin, TOB= tobramycin, AMP/AMR= ampi/amoxicillin, AMC = amoxycillin-clavulanic acid, TZP = piperacillin/tazobactam, CPM = cefepime, CTX/CRO = cefotaxime/ceftriaxone, FOX = cefoxitin, CXM = cefuroxime, CAZ = ceftazidime, CIP = ciprofloxacin, SXT = trimethoprim-sulfamethoxazole/co-trimoxazole, DOR = doripenem, ERT = ertapenem, IMP = imipenem, MEM = meropenem, TGC = tigecycline, NIT = nitrofurantoin.

### Genome characteristics

The genomic features of the sequenced isolates, such as coverage, GC content (%), genome size, L50, N50, and RNA count, are shown in Supplementary Table S1.

### Resistome analysis

Resistome analysis showed that all clinical *E. coli* isolates (100%) and 15 (88.2%) community isolates harboured β-lactamase ARGs. A total of seven *E. coli* isolates were identified as ESBL-positive (20% clinical; 23.5% community) (Table 2). Notably, we detected the *bla*_CTX−M_ gene in 10/15 clinical *E. coli* isolates but only 3/10 were identified as ESBL-producers by the VITEK 2 AES. In clinical isolates, we detected *bla*_CTX−M−15_ (8/15), *bla*_TEM−1B_ (4/15), *bla*_CTX−M−27_ (1/15), *bla*_CTX−M−231_ (1/15), *bla*_TEM−1 C_ (1/*15)*, and *bla*_OXA−1_ (1/15) ARGs. In addition, the carbapenemase *bla*_OXA−181_ ARG was detected in two clinical *E. coli* isolates (EC07 and EC19). Among community isolates, the most prevalent β-lactamase ARGs were *bla*_TEM−1B_ (12/17), followed by *bla*_CTX−M−15_ (2/17), *bla*_CTX−M−231_ (1/17), *bla*_CTX−M−27_ (1/17), *bla*_TEM−216_ (1/17), and *bla*_LAP−2_ (1/17). Fluoroquinolone *qnrS1* gene was the most detected (4/15 clinical; 6/17 community). The *aac(6’)-Ib-cr* gene was also identified in one clinical isolate (EC13). Several *E. coli* isolates (7/15 clinical; 2/17 community) exhibited phenotypic resistance to CIP despite the absence of corresponding ARGs, suggesting that resistance may be mediated by chromosomal mutations in the quinolone resistance-determining regions (QRDRs) of DNA gyrase (GyrA and GyrB) and topoisomerase IV (ParC and ParE). Fluoroquinolone-associated point mutations were analysed in all CIP resistant *E. coli* isolates. Mutations within the QRDRs of GyrA (S83L and D87N) and ParC (S80I and E84V) were the most frequently detected across isolates of both settings (Supplementary Table S2).

Trimethoprim ARGs in clinical isolates included *dfrA17* (12/15), *dfrA1* (1/15), dfrA5 (1/15), and *dfrA12* (1/15). Sulfonamide resistance was conferred either by *sul1* or *sul2*, or a combination of both in clinical and community isolates. Among community isolates, *dfrA14* (6/17) was most common, followed by *dfrA17* (3/17), *dfrA7* (3/17), *dfrA1* (2/17), and *dfrA5* (1/17). Aminoglycoside ARGs among clinical isolates included *aadA5* (10/15) as the most common, followed by *aph(3’’)-Ib* and *aph(6)-Id* (6/15 each). Other detected genes were *aph(3’)-Ia*, *aac(3)-IIe*, *aac(3)-IId*, *aadA1*, and *aadA3* (Table 2). In community isolates, *aph(3’’)-Ib* and *aph(6)-Id* were most prevalent (9/17 each), followed by *aadA1* (2/17) and *aadA5* (2/17). Other detected ARGs in clinical isolates included genes conferring resistance to macrolides [*mph(A)*], phenicols (*catA1* and *catB3*), and tetracycline [*tet(A)*]. Community isolates also harboured *catA1*, *mph(A)*, *tet(A)*, and *tet(B)* genes. Overall, comparative analyses of ARG prevalence revealed that most resistance genes did not differ significantly between clinical and community isolates after FDR correction; however, *aadA5*, *mph(A)*, *sul1*, and *dfrA17* were significantly more prevalent in clinical isolates (Supplementary Table S6).

Resistome analyses generally corresponded with phenotypic resistance. Two antibiograms were shared between clinical and community *E. coli* isolates, with AMP/AMX-SXT being the predominant shared pattern (2/15 clinical; 9/17 community), followed by AMP/AMX-CIP-SXT in one clinical (EC01) and one community (EC22) isolate (Table 2). Although these isolates were not genotypically identical, WGS revealed overlapping ARGs.

**Table 2:** Sequence types, antibiograms, ESBL production, resistome, efflux pump genes, and plasmid replicons of clinical and community *E. coli* isolates.

Isolate (MLST)	Setting	Antibiogram	ESBL +/-	Resistome	Efflux pump genes associated with resistance	Plasmid replicons
EC01 (ST3337)	Clinical	AMP/AMX-CIP-SXT	-	*bla*_TEM−1B_, *dfrA17*,* sul1*,* sul2*,* aadA5*,* aph(3’)-Ia*,* aph(3’’)-Ib*,* aph(6)-Id*,* catA1*,* mph(A)*,* tet(B)*	mdtH, kdpE, msbA, mdtN, mdtM, evgS, evgA, H-NS, marA, AcrA, cpxA, gadX, qacE∆1, leuO, CRP, rsmA, mdfA, baeR, AcrAB-TolC, tetR	IncQ1
EC02 (ST1193)	Clinical	AMP/AMX-CTX/CRO-CXM-CIP-SXT	-	*bla*_CTX−M−27_, *dfrA17*,* sul1*,* sul2*,* aadA5*,* aph(3’’)-Ib*,* aph(6)-Id*,* mph(A)*,* tet(A)*	leuO, H-NS, cpxA, gadW, evgA, qacE∆1, emrR, AcrA, rsmA, gadX, CRP, emrY, evgS, marA, kdpE, mdfA, AcrAB-TolC, baeR	Col(BS512), Col156, ColpVC, IncFIA, IncFIB(AP001918)
EC03 (ST131)	Clinical	AMP/AMX	-	*bla*_CTX−M−15_, *dfrA17*,* sul1*,* aadA5*,* mph(A)*	H-NS, leuO, gadW, cpxA, evgA, qacE∆1, kdpE, ermB, CRP, gadX, AcrA, evgS, baeR, AcrAB-TolC	IncFIA, IncFIB(AP001918), IncFII(pRSB107)
EC04 (ST38)	Clinical	AMP/AMX-CPM-CTX/CRO-CAZ-CXM-CIP-SXT	-	*bla*_CTX−M−15_, *dfrA5*,* dfrA12*,* sul1*,* sul2*,* aadA3*,* aac(3)-IIe*,* mph(A)*	marA, H-NS, emrR, evgA, cpxA, qacE∆1, leuO, CRP, kdpE, mdfA, evgS, gadX, AcrA, AcrAB-TolC, baeR	Col156, IncFIB(AP001918), IncFII
EC05 (ST38)	Clinical	AMP/AMX-SXT	-	*bla*_TEM−1 C_, *dfrA1*,* sul1*,* aadA1*,* aph(3’’)-Ib*,* aph(6)-Id*,* tet(A)*	marA, emrR, H-NS, evgA, cpxA, qacE∆1, leuO, CRP, rsmA, AcrA, evgS, gadX, AcrAB-TolC, baeR	IncFIA, IncFIB(AP001918), IncFIC(FII)
EC07 (ST3572)	Clinical	AMP/AMX-CTX/CRO-CXM-CIP-SXT	-	*bla*_OXA−181_, *dfrA17*,* sul1*,* sul2*,* aph(3’’)-Ib*,* aph(6)-Id*,* mph(A)*,* tet(A)*	marA, kdpE, emrY, evgA, evgS, AcrA, gadX, qacE∆1, H-NS, CRP, leuO, AcrAB-TolC, baeR	Col(BS512), Col(MG828), Col156, ColKP3, ColpEC648, IncFIA, IncM1
EC08 (ST69)	Clinical	AMP/AMX-SXT	-	*bla*_TEM−1B_, *dfrA17*,* sul2*,* aadA5*,* aph(3’’)-Ib*,* aph(6)-Id*,* mph(A)*,* tet(B)*	emrY, evgA, mdtN, marA, mdtM, H-NS, evgS, rsmA, CRP, gadX, leuO, kdpE, AcrA, AcrE, AcrAB-TolC, baeR, tetR	Col156, Col(MG828), IncFIB(AP001918), IncFII(29), IncQ1
EC09 (ST131)	Clinical	AMP/AMX-CTX/CRO-CAZ-CXM-CIP-GEN-SXT	+	*bla*_TEM−1B_, *bla*_CTX−M−15_, *dfrA17*,* sul1*,* sul2*,* aadA5*,* aac(3)-IId*,* aph(3’’)-Ib*,* aph(6)-Id*,* mph(A)*,* tet(A)*	leuO, cpxA, evgA, H-NS, gadW, AcrD, qacE∆1, mdfA, kdpE, CRP, TolC, emrY, evgS, marA, AcrA, gadX, baeR, AcrAB-TolC	Col156, ColpVC, IncFIA, IncFIB(AP001918), IncFII(pSE11), IncFII(pRSB107), IncQ1
EC10 (ST131)	Clinical	AMP/AMX-CTX/CRO-CXM-CIP-SXT	-	*bla*_CTX−M−15_, *dfrA17*,* sul1*,* aadA5*,* mph(A)*	H-NS, leuO, gadW, cpxA, evgA, qacE∆1, mdfA, kdpE, ermB, CRP, gadX, TolC, AcrA, evgS, baeR, AcrAB-TolC	IncFIA, IncFIB(AP001918), IncFII(pRSB107)
EC13 (ST131)	Clinical	AMP/AMX-CXM-CIP-SXT	-	*bla*_CTX−M−15_, *bla*_OXA−1_, *dfrA17*,* sul1*,* aadA5*,* aac(6’)-Ib-cr*,* catB3*,* mph(A)*,* tet(A)*	evgA, leuO, cpxA, gadW, H-NS, qacE∆1, mdfA, kdpE, AcrA, TolC, CRP, gadX, AcrAB-TolC, baeR	Col(BS512), IncFIA, IncFII
EC14 (ST10)	Clinical	AMP/AMX-CTX/CRO-CAZ-CXM-CIP-SXT	+	*bla*_CTX−M−15_, *dfrA17*,* sul1*,* aadA5*,* mph(A)*,* qnrS1*	H-NS, AcrA, kdpE, emrY, evgA, evgS, gadX, cpxA, qacE∆1, TolC, CRP, leuO, mdfA, baeR, AcrAB-TolC	Col(BS512), ColpVC, IncFII
EC15 (ST13823)	Clinical	AMP/AMX-CTX/CRO-CXM-SXT	-	*bla*_CTX−M−15_, *dfrA17*,* sul1*,* aadA5*,* qnrS1*	evgA, AcrA, marA, gadX, H-NS, qacE∆1, evgS, CRP, leuO, kdpE, cpxA, mdfA, baeR, AcrAB-TolC	IncFIB(AP001918)
EC16 (ST1193)	Clinical	AMP/AMX-CXM-CIP	-	*bla*_TEM−1B_, *bla*_CTX−M−231_, *mph(A)*,* qnrS1*	cpxA, H-NS, evgA, leuO, gadW, kdpE, emrB, CRP, evgS, AcrA, marA, gadX, baeR, AcrAB-TolC	Col(BS512), ColpVC, IncFIA, IncFIB(AP001918)
EC18 (ST13823)	Clinical	AMC-AMP/AMX-CPM-CTX/CRO-FOX-CAZ-CXM-CIP-TZP-SXT	+	*bla*_*C*TX−M−15_, *dfrA17*,* sul1*,* aadA5*,* qnrS1*	marA, evgA, AcrA, gadX, H-NS, qacE∆1, evgS, CRP, leuO, kdpE, cpxA, AcrAB-TolC, baeR	Col(MG828), ColpVC, IncFIB(AP001918), pXuzhou21
EC19 (ST3572)	Clinical	AMC-AMP/AMX-CTX/CRO-FOX-CXM-CIP-GEN-TZP-SXT	-	*bla*_OXA−181_, *dfrA17*,* sul1*,* mph(A)*,* tet(A)*	marA, emrY, evgA, evgS, AcrA, kdpE, H-NS, cpxA, gadX, AcrD, qacE∆1, leuO, CRP, baeR, AcrAB-TolC	ColKP3, IncM1
EC21 (ST1722)	Community	AMP/AMX-CXM-CPM-CTX/CRO-CAZ-AMC-SXT-TZP	+	*bla*_CTX−M−15_, *bla*_TEM−216_, *dfrA1*,* sul1*,* aadA1*,* qnrS1*	kdpE, marA, emrR, cpxA, evgA, H-NS, qacE∆1, leuO, AcrA, TolC, CRP, gadX, evgS, AcrAB-TolC, baeR	Col(BS512), Col(MG828), IncB/O/K/Z
EC22 (ST1193)	Community	AMP/AMX-CIP-SXT	-	*bla*_TEM−1B_, *dfrA17*,* sul2*,* aph(3’’)-Ib*,* aph(6)-Id*,* mph(A)*,* tet(B)*	leuO, gadW, H-NS, evgA, cpxA, kdpE, mdfA, AcrA, gadX, emrY, evgS, marA, baeR, AcrAB-TolC	Col(BS512), Col156, IncFIA, IncFIB(AP001918), IncQ1
EC23 (ST10)	Community	AMP/AMX-SXT	-	*bla*_TEM−1B_, *dfrA14*,* sul2*,* aph(3’’)-Ib*,* aph(6)-Id*,* tet(A)*	AcrA, kdpE, cpxA, marA, emrY, evgA, H-NS, leuO, CRP, mdfA, evgS, AcrAB-TolC, baeR	Col(MG828), IncFII, IncFII(pCoo), IncFII(pHN7A8), IncX1
EC24 (ST1380)	Community	SXT	-	*dfrA14*,* sul2*,* aph(3’’)-Ib*,* aph(6)-Id*,* tet(A)*	marA, emrY, evgA, H-NS, gadX, kdpE, evgS, leuO, AcrA, mdfA, gadX, AcrAB-TolC, baeR	Col(MG828), IncB/O/K/Z, IncFIB(AP001918), IncFII, IncFII(pCoo), IncFII(pHN7A8), IncFII(pRSB107), IncX1
EC25 (ST10)	Community	AMP/AMX-SXT	-	*bla*_TEM−1B_, *dfrA14*,* sul2*,* aph(3’’)-Ib*,* aph(6)-Id*,* tet(A)*	AcrA, kdpE, cpxA, marA, emrY, evgA, H-NS, leuO, CRP, mdfA, evgS, AcrAB-TolC, baeR	Col(MG828), IncFII, IncFII(pCoo), IncFII(pHN7A8), IncX1
EC26 (ST131)	Community	AMP/AMX-CXM-CPM-CTX/CRO-CAZ-CIP-AMC-TZP	+	*bla*_CTX−M−27_	evgA, gadW, leuO, cpxA, H-NS, emrY, evgS, marA, CRP, gadX, TolC, kdpE, AcrA, mdfA, rsmA, baeR, AcrAB-TolC	Col(MG828), IncFIA, IncFIB(AP001918),
EC30 (ST2758)	Community	AMP/AMX-SXT	-	*bla*_LAP−2_, *bla*_TEM−1B_, *dfrA14*,* sul2*,* aph(3’’)-Ib*,* aph(6)-Id*,* qnrS1*	evgA, cpxA, marA, gadX, H-NS, AcrA, leuO, CRP, mdfA, kdpE, TolC, emrY, evgS, baeR, AcrAB-TolC	IncFIB(K), IncFII
EC31 (ST2852)	Community	AMP/AMX-SXT	-	*bla*_TEM−1B_, *dfrA14*,* sul2*,* tet(A)*,* qnrS1*	H-NS, evgA, AcrA, marA, cpxA, emrY, evgS, CRP, gadX, leuO, kdpE, mdfA, baeR, AcrAB-TolC	IncY
EC32 (ST382)	Community	AMP/AMX-SXT	-	*bla*_TEM−1B_, *dfrA7*,* sul2*,* aph(3’’)-Ib*,* aph(6)-Id*,* tet(B)*	evgA, AcrA, H-NS, marA, gadX, qacE∆1, leuO, emrY, evgS, kdpE, CRP, cpxA, baeR, AcrAB-TolC	IncFIB(AP001918), IncQ1
EC33 (ST336)	Community	MEM-TZP	-	*bla*_TEM−1B_, *dfrA14*,* sul2*,* tet(A)*,* qnrS1*	H-NS, marA, evgA, gadX, leuO, kdpE, CRP, rsmA, AcrAB-TolC	Col(BS512), IncFII(pRSB107), IncY
EC35 (ST2178)	Community	AMP/AMX-SXT	-	*bla*_TEM−1B_, *dfrA7*,* sul2*,* aph(3’’)-Ib*,* aph(6)-Id*	marA, evgA, H-NS, AcrA, cpxA, qacE∆1, gadX, evgS, leuO, kdpE, mdfA, CRP, baeR, AcrAB-TolC	IncFIB(AP001918), IncFII
EC36 (ST101)	Community	SXT	-	*dfrA1*,* sul1*,* aadA1*,* aph(6)-Id*,* tet(A)*	marA, evgA, AcrA, H-NS, cpxA, qacE∆1, emrY, evgS, mdfA, leuO, kdpE, CRP, gadX, AcrAB-TolC, baeR	IncFIB(AP001918), IncFII(pCoo), IncX1
EC37 (ST131)	Community	AMP/AMX-SXT	-	*bla*_TEM−1B_, *dfrA17*,* sul1*,* sul2*,* aadA5*,* aph(3’’)-Ib*,* mph(A)*	evgA, H-NS, gadW, leuO, cpxA, qacE∆1, mdfA, kdpE, evgS, AcrA, TolC, CRP, AcrAB-TolC, baeR	Col156, IncFIB(AP001918), IncFII(29), IncQ1
EC38 (ST4406)	Community	AMP/AMX-SXT	-	*bla*_TEM−1B_, *dfrA7*,* sul2*,* aph(3’’)-Ib*,* aph(6)-Id*,* catA1*,* tet(A)*	kdpE, qacE∆1, H-NS, cpxA, marA, AcrA, leuO, emrY, evgA, evgS, CRP, gadX, mdfA, TolC, AcrAB-TolC, baeR	IncFII(29)
EC39 (ST3570)	Community	AMP/AMX-SXT	-	*bla*_TEM−1B_, *dfrA5*,* sul1*,* catA1*	marA, AcrA, evgA, cpxA, H-NS, qacE∆1, evgS, leuO, gadX, CRP, mdfA, kdpE, baeR, AcrAB-TolC	Col(BS512), IncB/O/K/Z, IncFIB(AP001918), IncFII(pCoo)
EC40 (ST13823)	Community	AMP/AMX-CXM-CPM-CTX/CRO-CAZ-AMC-SXT-TZP	+	*bla*_CTX−M−15_, *dfrA17*,* sul1*,* aadA5*,* qnrS1*	evgA, AcrA, marA, gadX, H-NS, qacE∆1, evgS, CRP, leuO, kdpE, cpxA, mdfA, baeR, AcrAB-TolC	IncFIB(AP001918)
EC41 (ST210)	Community	AMP/AMX-CXM-CPM-CTX/CRO-CAZ-CIP-AMC-TZP	+	*bla*_TEM−1B_, *bla*_CTX−M−231_, *mph(A)*,* qnrS1*	AcrA, evgA, marA, H-NS, CRP, gadX, leuO, evgS, mdfA, kdpE, ermB, AcrAB-TolC, baeR	IncB/O/K/Z, IncFII

Key: AMK = amikacin, GEN = gentamicin, TOB = tobramycin, AMP/AMR = ampi/amoxicillin, AMC = amoxycillin-clavulanic acid, TZP = piperacillin/tazobactam, CPM = cefepime, CTX/CRO = cefotaxime/ceftriaxone, FOX = cefoxitin, CXM = cefuroxime, CAZ = ceftazidime, CIP = ciprofloxacin, SXT = trimethoprim-sulfamethoxazole/co-trimoxazole, DOR = doripenem, ERT = ertapenem, IMP = imipenem, MEM = meropenem, TGC = tigecycline, NIT = nitrofurantoin.

Among *K. pneumoniae* isolates, one community isolate (EC27) was identified as an ESBL-producer (Table 3). Clinical isolates predominantly harboured the *bla*_CTX−M−15_ gene (5/6), frequently co-occurring with *bla*_SHV−1_, *bla*_SHV−11_, and *bla*_TEM−1B_. One isolate (KP46) also harboured the carbapenemase *bla*_NDM−5_ and *bla*_OXA−181_ genes, while another (KP51) carried *bla*_TEM−34_ and *bla*_SHV−75_. The *bla*_SHV−1_, *bla*_SHV−11_, *bla*_SHV−27_, and *bla*_SHV−61_ were detected among community isolates. Two community isolates (KP57 and KP59) carried the *bla*_TEM−1_ gene, and one isolate (KP53) carried the *bla*_LAP−2_ gene. No QRDR mutations were detected in CIP resistant *K. pneumoniae* isolates. Instead, resistance may have been mediated by *aac(6’)-Ib-cr* (4/6), frequently co-occurring with rifampicin (*arr-3*) genes, followed by *qnrB6* (3/6) and *qnrS1* (1/6) genes in clinical isolates (Table 3). In community isolates, *qnrS1* was identified in two isolates.

Trimethoprim ARGs detected in clinical isolates included *dfrA12*, *dfrA14*, and *dfrA27*, whereas community isolates harboured *dfrA5*, *dfrA8*, and *dfrA14*. The *sul2* gene was the most prevalent sulfonamide ARG among all *K. pneumoniae* isolates, while *sul1* was detected in four clinical isolates and one community isolate. Moreover, a *sul3* gene was identified in one community isolate (KP53). *aph(3’’)-Ib*, *aph(6)-Id*, *aac(3)-IIe*, *aadA2* and *aadA16* aminoglycoside ARGs were detected in clinical *K. pneumoniae* isolates. Community isolates harboured *aph(3’)-Ia*, *aph(3’’)-Ib*, *aph(6)-Id* and *aadA1* ARGs. Fosfomycin (*fosA5*) resistance genes were detected in all *K. pneumoniae* isolates. Additional resistance genes were detected in both clinical and community isolates. One clinical isolate carried *catA2* and *tet(D)*, conferring resistance to chloramphenicol and tetracyclines, while another carried *mph(A)*, conferring resistance to macrolides. Among community isolates, *mph(A)* was detected in one, and tetracycline [*tet(A)* or *tet(D)*] in two. Comparative analyses showed that no differences in ARGs remained statistically significant after FDR correction, although several genes differed in prevalence between clinical and community isolates at the unadjusted p-value level (Supplementary Table S7).

**Table 3:** Sequence types, antibiograms, ESBL production, resistome, efflux pump genes, and plasmid replicons of clinical and community *K. pneumoniae* isolates.

Isolate (MLST)	Setting	Antibiogram	ESBL +/-	Resistome	Efflux pump genes associated with resistance	Plasmid replicons
KP43 (ST101)	Clinical	AMP/AMX-FOX	-	*bla*_SHV−1_, *aph(3’’)-Ib*,* aph(6)-Id*,* fosA5*	oqxA, oqxB20, H-NS, CRP, adeF, leuO, msbA, rsmA, KpnH, KpnG, KpnF, KpnE, emrA, emrB, emrR, adeF, acrA, marA, mdtN, AcrAB-TolC, baeR	IncFIA(HI1), IncFIB(K)(pCAV1099-114), IncFII(pKPX1), repB(R1701)
KP45 (ST348)	Clinical	AMP/AMX-CPM-CTX/CRO-CAZ-CXM-SXT	-	*bla*_CTX−M−15_, *bla*_SHV−11_, *dfrA14*,* sul2*,* aph(3’’)-Ib*,* aph(6)-Id*,* catA2*,* fosA5*,* tet(D)*	oqxA, oqxB25, KpnF, KpnG, KpnH, KpnE, LptD, adeF, emrA, emrB, emrR, CRP, msbA, acrA, rsmA, H-NS, marA, leuO, AcrAB-TolC, baeR	IncFIB(K), IncFII(K)
KP46 (ST17)	Clinical	AMC-AMP/AMX-CTX/CRO-FOX-CAZ-CXM-CIP-GEN-IMP-MEM-TZP-SXT	-	*bla*_CTX−M−15_, *bla*_NDM−5_, *bla*_OXA−181_, *bla*_SHV−11_, *bla*_TEM−1B_, *dfrA12*,* dfrA27*,* sul1*,* sul2*,* aadA2*,* aadA16*,* aac(3)-IIe*,* aac(6’)-Ib-cr*,* aph(3’’)-Ib*,* aph(6)-Id*,* arr-3*,* fosA5*,* mph(A)*,* qnrS1*	KpnE, KpnF, KpnG, KpnH, oqxA, oqxB25, LptD, qacE∆1, H-NS, msbA, adeF, acrD, acrA, emrA, ermB, emrR, rsmA, leuO, CRP, AcrAB-TolC, baeR	ColKP3, IncFIA(HI1), IncFIB(K), IncFII, IncFII(K), IncR, IncX3
KP47 (ST607)	Clinical	AMC-AMP/AMX-CTX/CRO-FOX-CAZ-CXM-CIP-GEN-NIT-TZP-SXT	-	*bla*_CTX−M−15_, *bla*_SHV−1_, *bla*_TEM−1B_, *dfrA27*,* sul1*,* sul2*,* aadA16*,* aac(3)-IIe*,* aac(6’)-Ib-cr*,* aph(3’’)-Ib*,* aph(6)-Id*,* arr-3*,* fosA5*,* qnrB6*	oqxA, oqxB19, KpnF, KpnG, KpnH, KpnE, LptD, acrD, qacE∆1, CRP, adeF, emrA, emrB, emrR, rsmA, marA, H-NS, acrA, leuO, msbA, AcrAB-TolC, baeR	ColRNAI, IncFIA(HI1), IncFIB(K), IncFII(K), IncR
KP48 (ST607)	Clinical	AMC-AMP/AMX-CTX/CRO-CAZ-CXM-CIP-GEN-NIT-TZP-SXT	-	*bla*_CTX−M−15_, *bla*_SHV−1_, *bla*_TEM−1B_, *dfrA27*,* sul1*,* sul2*,* aadA16*,* aac(3)-IIe*,* aac(6’)-Ib-cr*,* aph(3’’)-Ib*,* aph(6)-Id*,* arr-3*,* fosA5*,* qnrB6*	oqxA, oqxB19, LptD, KpnF, KpnG, KpnH, KpnE, acrD, qacE∆1, adeF, emrA, emrB, emrR, rsmA, CRP, H-NS, acrA, leuO, marA, msbA, baeR, AcrAB-TolC	ColRNAI, IncFIA(HI1), IncFIB(K), IncFII(K), IncR
KP51 (ST133)	Clinical	AMC-AMP/AMX-CPM-CTX/CRO-CAZ-CXM-CIP-GEN-TZP-SXT	-	*bla*_CTX−M−15_, *bla*_SHV−75_, *bla*_TEM−34_, *dfrA27*,* sul1*,* sul2*,* aadA16*,* aac(3)-IIe*,* aac(6’)-Ib-cr*,* aph(3’’)-Ib*,* aph(6)-Id*,* arr-3*,* fosA5*,* mph(A)*,* qnrB6*	LptD, oqxA, oqxB14, KpnE, KpnF, KpnH, KpnG, acrD, qacE∆1, adeF, leuO, acrA, rsmA, H-NS, CRP, emrA, emrB, emrR, msbA, AcrAB-TolC, baeR	Col(pHAD28), IncFIA(HI1), IncFII, IncFII(pBK30683), IncI2, IncR
EC27 (ST716)	Community	AMP/AMX-CXM-CPM-CTX/CRO-CAZ-AMC-TZP	+	*bla*_SHV−27_, *dfrA5*,* sul1*,* fosA5*	oqxA, oqxB19, KpnF, KpnG, KpnH, KpnE, LptD, qacE∆1, adeF, H-NS, emrA, emrB, emrR, rsmA, msbA, acrA, marA, leuO, CRP, baeR, AcrAB-TolC	IncFIB(pQil), repB
KP53 (ST193)	Community	AMP/AMX-CIP-TGC	-	*bla*_SHV−61_, *bla*_LAP−2_, *sul2*,* sul3*,* aadA1*,* aph(3’)-Ia*,* fosA5*,* qnrS1*,* tet(A)*	oqxA, oqxB25, cmlA1, KpnF, KpnG, KpnH, KpnE, mef(B), H-NS, adeF, emrA, emrB, emrR, rsmA, leuO, LptD, qacL, msbA, marA, CRP, acrA, baeR, AcrAB-TolC	IncFIB(pNDM-Mar)
KP54 (ST5396)	Community	AMP/AMX-TGC	-	*bla*_SHV−1_, *fosA5*	KpnF, KpnE, KpnG, KpnH, oqxA, oqxB19, LptD, marA, CRP, msbA, adeF, emrA, emrB, emrR, rsmA, leuO, acrA, AcrAB-TolC, baeR	None
KP55 (ST17)	Community	AMP/AMX-TGC	-	*bla*_SHV−11_, *fosA5*	oqxA, oqxB25, LptD, KpnE, KpnF, KpnG, KpnH, H-NS, adeF, emrA, emrB, emrR, rsmA, msbA, acrA, CRP, leuO, AcrAB-TolC, baeR	IncFIB(K), IncFII(K), IncR, repB(R1701)
KP56 (ST37)	Community	AMP/AMX-TGC	-	*bla*_SHV−11_, *fosA5*	KpnF, KpnE, KpnG, KpnH, oqxA, oqxB, LptD, marA, rsmA, H-NS, msbA, CRP, acrA, AcrAB-TolC, baeR	Col440I, IncFIB(K), IncFII(K), IncR
KP57 (ST45)	Community	AMP/AMX-AMC-SXT-TZP	-	*bla*_SHV−1_, *bla*_TEM−1B_, *dfrA14*,* sul2*,* aph(3’’)-Ib*,* aph(6)-Id*,* fosA5*,* mph(A)*	LptD, KpnF, KpnG, KpnH, KpnE, KpnF, H-NS, emrR, rsmA, marA, acrA, CRP, leuO, msbA, oqxA11, oqxB, adeF	Col(pHAD28), IncFIB(K), IncFII(K), IncQ1
KP58 (ST873)	Community	AMP/AMX-SXT	-	*bla*_SHV−27_, *dfrA8*,* sul2*,* fosA5*,* tet(D)*	KpnF, KpnE, KpnG, KpnH, LptD, marA, msbA, emrR, rsmA, adeF, acrA, H-NS, leuO, CRP, oqxA11, oqxB19, AcrAB-TolC, baeR	IncFIB(K), IncFII(K), IncR
KP59 (ST1863)	Community	AMP/AMX-CIP	-	*bla*_SHV−1_,*bla*_TEM−1B_, *fosA5*,* qnrS1*	KpnF, KpnE, KpnG, KpnH, oqxA, OqxB, LptD, H-NS, marA, CRP, adeF, emrA, emrB, emrR, rsmA,	IncN
					leuO, acrA, msbA, baeR, AcrAB-TolC	

Key: AMK = amikacin, GEN = gentamicin, TOB = tobramycin, AMP/AMR = ampi/amoxicillin, AMC = amoxycillin-clavulanic acid, TZP = piperacillin/tazobactam, CPM = cefepime, CTX/CRO = cefotaxime/ceftriaxone, FOX = cefoxitin, CXM = cefuroxime, CAZ = ceftazidime, CIP= ciprofloxacin, SXT = trimethoprim-sulfamethoxazole/co-trimoxazole, DOR= doripenem, ERT = ertapenem, IMP = imipenem, MEM = meropenem, TGC = tigecycline, NIT = nitrofurantoin.

### Mobilome analysis and associated ARGs

The IncF plasmid replicon family (IncFIA, IncFIB, IncFII, and IncFIC) was the most prevalent, detected in 87% of isolates. IncFIB was the most common replicon among *E. coli* (20/32, 62.5%) (Table 2) and *K. pneumoniae* (11/14, 78.6%) isolates (Table 3). IncFIA was detected in 8/15 clinical and 2/17 community *E. coli* isolates, as well as in 5/6 clinical *K. pneumoniae isolates*, but none in community *K. pneumoniae* isolates. IncFII plasmids were detected in *E. coli* (7/15, 46.7% clinical; 11/17, 64.7% community) and *K. pneumoniae* (6/6, 100% clinical; 4/8, 50% community) isolates. IncFIC(FII) was only detected in one clinical *E. coli* isolate (EC05). The IncR plasmid replicon was present in clinical (4/6) and community (3/8) *K. pneumoniae* isolates. Clinical *E. coli* isolates exhibited a broader range of Col plasmid replicons, including Col(MG828), which was detected in isolates from both settings. Col plasmid replicons were also detected in *K. pneumoniae* isolates; however, none were shared between clinical and community settings. In both *E. coli* and *K. pneumoniae*, Col plasmid replicons commonly co-occurred with IncF plasmids. Notably, we detected ColKP3 replicons in all *bla*_OXA−181_-harbouring clinical *E. coli* and *K. pneumoniae* isolates.

Synteny analysis revealed that most ARGs were co-localized with class 1 integrons, ISs, and Tn*3* on the same contig, with most ARGs and MGEs encoded on plasmid-associated contigs (Supplementary Table S3 and S4). The *bla*_TEM−1_ was commonly found with IS*1* and Tn*3* in clinical and community *E. coli* isolates. IS*Kpn19* and IS*3000* commonly co-localized with the *bla*_OXA−181_ ARG, while *bla*_CTX−M−15_ was co-localized with Tn*3* and IS*3* in most clinical and community *E. coli* isolates along with IS*Kpn19* and *qnrS1* on the same contig. The *bla*_CTX−M−27_ ARG was associated with IS*5* in both clinical (EC02) and community (EC26) isolates, while *bla*_CTX−M−231_ (EC16 and EC41) was associated with IS*26*. In community isolates, *aph(3’’)-Ib* and *aph(6)-Id* were associated with a range ISs (IS*5075*, IS*91*, IS*26* and IS*903B*). In contrast, *aph(3’’)-Ib* and *aph(6)-Id* in clinical *E. coli* isolates were not commonly associated with MGEs (Supplementary Table S3). All detected *qnrS1* ARGs in *E. coli* isolates were co-localized with Tn*3*, IS*Kpn19*, and IS*3*. Among *K. pneumoniae* clinical isolates, *bla*_TEM−1_ was predominantly associated with IS*Ecp1*, and *bla*_TEM−34_ showed a similar association, whereas *bla*_TEM−1_ from community isolates was associated with IS*6*. Most *bla*_CTX−M−15_ ARGs were co-localized with IS*Ecp1* (Supplementary Table S4). The *bla*_NDM−5_ ARG was associated with IS*30* and IS*CR1* and *bla*_OXA−181_ with Tn*3* and IS*3000* in KP46. *aph(3’’)-Ib* and *aph(6)-Id* in clinical isolates were co-localized with at least one insertion sequence (IS*91* or IS*Pa38*) and a *sul2* gene. In KP48, *aph(6)-Id* was only co-localized with IS*91*. Aminoglycoside ARGs in community *K. pneumoniae* isolates were not associated with MGEs. Among *K. pneumoniae* community isolates, *sul2* was associated with IS*Vsa3*, while *sul3* was co-localized with an *intI1* gene, along with *mef(B)*, IS*256*, *qacL*, *aadA1*, *cmlA1*, and Tn*As1*. All detected *qnrB6* and *qnrS1* ARGs were associated with IS*CR1* and IS*Kpn19*, respectively.

Only class 1 integrons (*intI1*) were detected among the isolates, present in 84.4% *E. coli* and 57.1% *K. pneumoniae* isolates (Table 4). The In54 integron was predominantly detected in clinical *E. coli* isolates, particularly those of ST131, and was associated with the *dfrA17*-*aadA5* gene cassette array. The most prevalent integron in community *E. coli* isolates was In191, containing the *dfrA14* gene cassette. In clinical *K. pneumoniae*, In1021 was the predominant integron harbouring the *aacA4cr*-*arr*-*3-dfrA27*-*aadA16* gene cassette array. Among community *K. pneumoniae*, the occurrence of class 1 integrons was less frequent, with only two isolates harbouring In13 (associated with *dfrA5*) and In191.

**Table 4:** Class 1 integrons and gene cassettes found in clinical and community *E. coli* and *K. pneumoniae* isolates.

Sample ID	Species	Setting	Integron class	Integron	Gene cassette arrays			
					GC1	GC2	GC3	GC4
EC01	*E. coli*	Clinical	IntI1	In54	*dfrA17*	*aadA5*	*−*	*−*
EC02	*E. coli*	Clinical	IntI1	In54	*dfrA17*	*aadA5*	*−*	*−*
EC03	*E. coli*	Clinical	IntI1	In54	*dfrA17*	*aadA5*	*−*	*−*
EC05	*E. coli*	Clinical	IntI1	−	*−*	*−*	*−*	*−*
EC07	*E. coli*	Clinical	IntI1	In987	*dfrA17*	*−*	*−*	*−*
EC08	*E. coli*	Clinical	IntI1	In987	*dfrA17*	*−*	*−*	*−*
EC09	*E. coli*	Clinical	IntI1	In54	*dfrA17*	*aadA5*	*−*	*−*
EC10	*E. coli*	Clinical	IntI1	In54	*dfrA17*	*aadA5*	*−*	*−*
EC13	*E. coli*	Clinical	IntI1	In54	*dfrA17*	*aadA5*	*−*	*−*
EC14	*E. coli*	Clinical	IntI1	In54	*dfrA17*	*aadA5*	*−*	*−*
EC15	*E. coli*	Clinical	IntI1	In54	*dfrA17*	*aadA5*	*−*	*−*
EC18	*E. coli*	Clinical	IntI1	In54	*dfrA17*	*aadA5*	*−*	*−*
EC19	*E. coli*	Clinical	IntI1	In987	*dfrA17*	*−*	*−*	*−*
EC21	*E. coli*	Community	IntI1	In183	*dfrA1*	*−*	*−*	*−*
EC22	*E. coli*	Community	IntI1	In987	*dfrA17*	*−*	*−*	*−*
EC23	*E. coli*	Community	IntI1	In191	*dfrA14*	*−*	*−*	*−*
EC25	*E. coli*	Community	IntI1	In191	*dfrA14*	*−*	*−*	*−*
EC30	*E. coli*	Community	IntI1	In191	*dfrA14*	*−*	*−*	*−*
EC31	*E. coli*	Community	IntI1	In191	*dfrA14*	*−*	*−*	*−*
EC32	*E. coli*	Community	IntI1	In22	*dfrA7*	*−*	*−*	*−*
EC33	*E. coli*	Community	IntI1	In191	*dfrA14*	*−*	*−*	*−*
EC35	*E. coli*	Community	IntI1	In22	*dfrA7*	*−*	*−*	*−*
EC36	*E. coli*	Community	IntI1	In183	*dfrA1*	*−*	*−*	*−*
EC37	*E. coli*	Community	IntI1	In54	*dfrA17*	*aadA5*	*−*	*−*
EC38	*E. coli*	Community	IntI1	In22	*dfrA7*	*−*	*−*	*−*
EC39	*E. coli*	Community	IntI1	In13	*dfrA5*	*−*	*−*	*−*
EC40	*E. coli*	Community	IntI1	In54	*dfrA17*	*aadA5*	*−*	*−*
KP45	*K. pneumoniae*	Clinical	IntI1	In191	*dfrA14*	*−*	*−*	*−*
KP46	*K. pneumoniae*	Clinical	IntI1	In1021	*aacA4cr*	*arr-3*	*dfrA27*	*aadA16*
KP47	*K. pneumoniae*	Clinical	IntI1	In1021	*aacA4cr*	*arr-3*	*dfrA27*	*aadA16*
KP48	*K. pneumoniae*	Clinical	IntI1	In1021	*aacA4cr*	*arr-3*	*dfrA27*	*aadA16*
KP51	*K. pneumoniae*	Clinical	IntI1	In1021	*aacA4cr*	*arr-3*	*dfrA27*	*aadA16*
EC27	*K. pneumoniae*	Community	IntI1	In13	*dfrA5*	*−*	*−*	*−*
KP53	*K. pneumoniae*	Community	IntI1	−	*−*	*−*	*−*	*−*
KP57	*K. pneumoniae*	Community	IntI1	In191	*dfrA14*	*−*	*−*	*−*

*aacA4cr* is synonymous with *aac(6’)-br-cr* on the INTEGRALL database.

### Sequence types and phylogenomic relationships

Overall, community isolates showed a higher clonal diversity compared to clinical isolates. Among all *E. coli* isolates, the most frequently detected sequence types (STs) were ST131, ST10, and ST1193, each identified in both clinical and community settings. ST131 predominated among clinical isolates (*n* = 4) and was also detected in two community isolates. ST10 was present in one clinical and two community isolates, while ST1193 was detected in two clinical and one community isolate (Table 2). Other sequence types detected in clinical isolates included ST38, ST1193, ST3572, and ST13823 (*n* = 2 each), as well as singletons ST69 and ST3337. Community *E. coli* isolates included several singletons (ST1193, ST1380, ST1722, ST2758, ST2852, ST382, ST336, ST2178, ST101, ST4406, ST3570, ST13823, and ST210). Despite differences in their antibiograms and plasmid profiles, the ST13823 isolates shared an identical resistome and the same synteny with MGEs, and all carried the IncFIB plasmid replicon. Similarly, community isolates belonging to ST10 had the same antibiogram, resistome content with the same MGE associations, and plasmid replicon profiles. Clinical *K. pneumoniae* isolates belonged to five different STs, with ST607 identified in two isolates, while ST101, ST348, ST17, and ST133 were singletons (Table 3). The ST607 isolates shared similar antibiograms and displayed the same associations with MGEs and plasmid replicons. In *K. pneumoniae* community isolates, all eight isolates were assigned to distinct STs (ST17, ST37, ST45, ST193, ST5396, ST716, ST873, and ST1863).

**Fig. 1:**
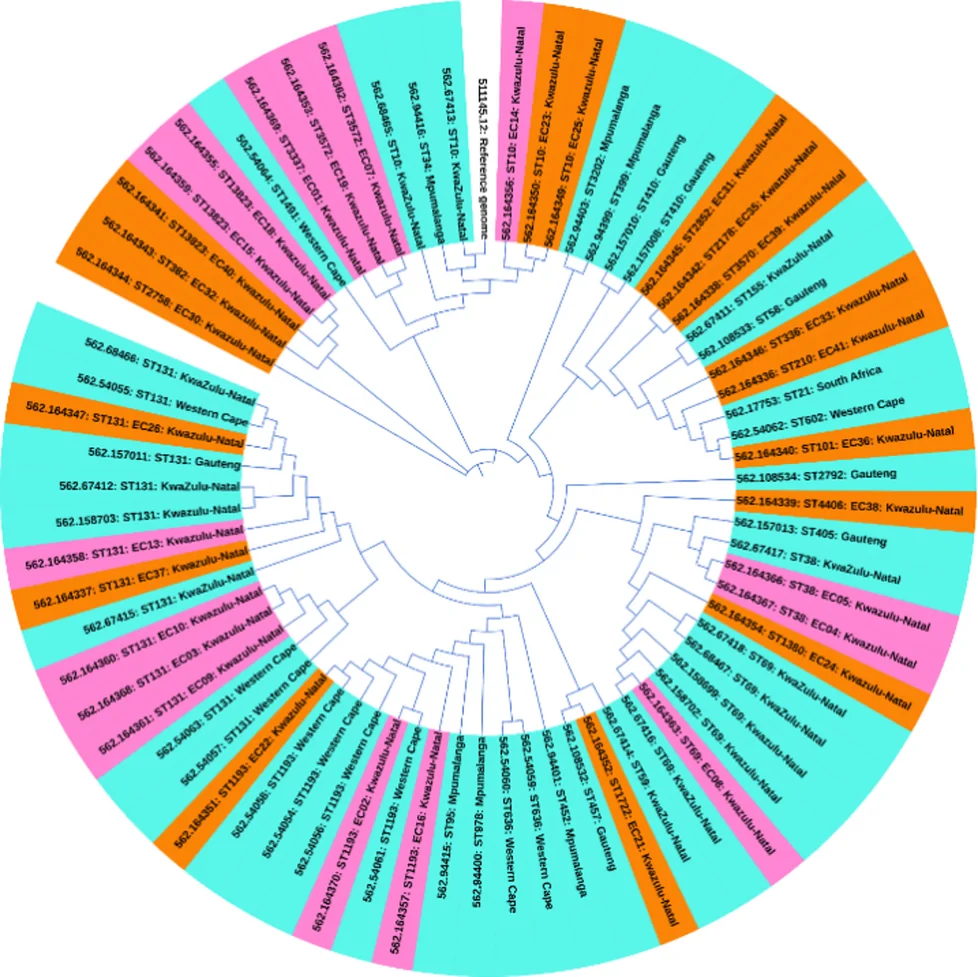
A maximum-likelihood phylogenetic tree illustrating the relationship between *E. coli* community (highlighted in orange) and clinical (highlighted in pink) isolates from this study and other South African isolates from diverse clinical sources (highlighted in turquoise). The *E. coli* K12 MG1655 strain was used as a reference genome.

**Fig. 2:**
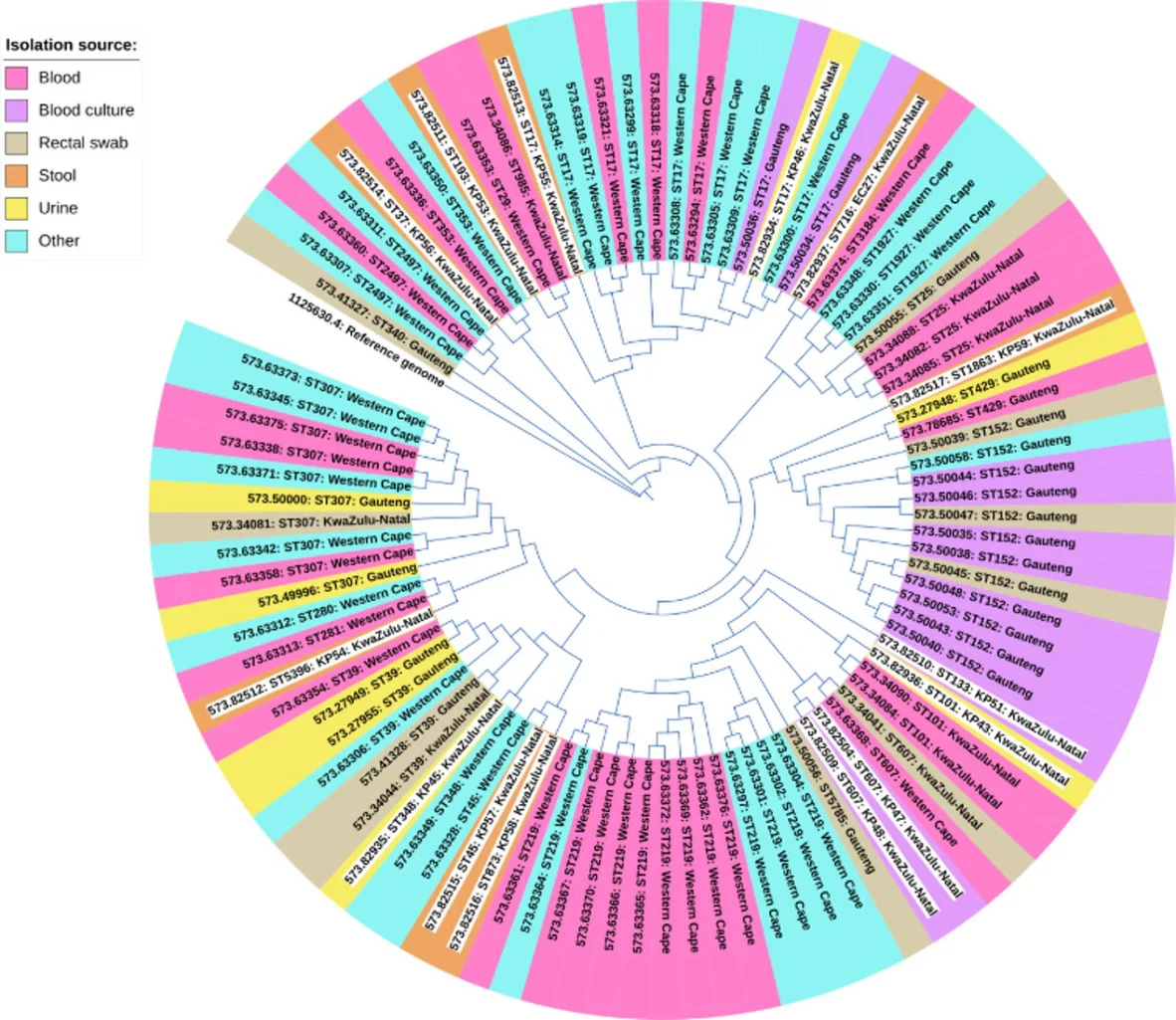
A maximum-likelihood phylogenetic tree illustrating the relationship between *K. pneumoniae* isolates from the community (stool) and clinical isolates (blood culture and urine highlighted in white) of this study, alongside other South African isolates from diverse clinical sources (blood, blood culture, rectal swab, urine and others). The *K. pneumoniae* HS11286 strain was used as a reference genome.

Phylogenetic analysis revealed clustering of clinical and community isolates of this study with other South African isolates from Gauteng, Mpumalanga, Western Cape and KwaZulu-Natal based on their STs. Among *E. coli* isolates, all clinical isolates clustered with community isolates (Fig. 1). Notably, six community isolates (EC35, EC39, EC41, EC31, EC33, and EC36) formed a distinct clade together with clinical isolates from the provinces listed above. In contrast, clinical *K. pneumoniae* isolates showed less frequent clustering with community isolates. KP46 clustered with community isolates KP53 and KP55 along with other clinical isolates from Western Cape and Gauteng (Fig. 2). Similarly, KP45 clustered with community isolates KP57 and KP58 along with other clinical isolates from Gauteng, Western Cape and KwaZulu-Natal. The remaining clinical (KP51, KP53, KP47 and KP48) and community isolates (KP56, EC27, KP59 and KP54) formed clades with other clinical isolates from Gauteng, Western Cape and KwaZulu-Natal.

## Discussion

There is a paucity of genomic data from healthy community dwellers in South Africa, which limits our understanding of the community resistome and its associated mobilome. This study provides a comparative genomic snapshot into the resistome, mobilome, clonalities, and phylogenies of *E. coli* and *K. pneumoniae* isolates obtained from stool samples of healthy community dwellers in uMgungundlovu alongside clinical isolates collected from a nearby hospital. Resistome analyses revealed a diverse array of ARGs conferring resistance to β-lactams, aminoglycosides, fluoroquinolones, trimethoprim-sulfamethoxazole, macrolides, fosfomycin, and tetracyclines. Several ARGs were located on plasmids in both community and clinical isolates, with many associated with transposons, integrons and insertion sequences. Notably, aminoglycoside ARGs detected in community *K. pneumoniae* isolates were not associated with any MGEs other than the plasmid replicons that were detected at the isolate level.

Both clinical and community *E. coli* isolates showed resistance to largely similar antibiotics, although most individual antibiograms differed. Genotypically, isolates were not identical; however, resistome analysis revealed overlapping key ARGs across both settings. High SXT resistance was observed in both clinical (86.7%) and community (82.4%) isolates, consistent with reports of ≥ 70% resistance in both healthy humans and clinical *E. coli* across Africa^25–32^. SXT prophylaxis is used to prevent opportunistic infections and malaria in people living with HIV^33^, and uMgungundlovu has a high HIV prevalence rate^19^, which can explain the high prevalence of SXT resistance in both clinical and community isolates. Furthermore, CIP and CXM resistance were also high among clinical *E. coli* isolates (73.3% each), consistent with findings from Zambia (67.9% CIP; 62% CXM)^31^. In South Africa, CIP is widely used at both primary healthcare and hospital levels for common infections, including urinary tract infections (UTIs), which may contribute to local selective pressure^34,35^. Naidoo et al., (2023)^36^, reported that *E. coli* accounted for 53% of uropathogens at a regional hospital in KwaZulu-Natal, with 38% of isolates resistant to CIP. Notably, most CIP and CXM resistant isolates in this study were recovered from urine specimens. Community *E. coli* isolates demonstrated a CXM and CIP resistance prevalence of 23.5% and 17.6%, respectively, which are comparable to previous community-based studies reporting concurrent CXM and CIP resistance, including 14% CXM and 19.3% CIP in commensal *E. coli* in China^37^ and 22.4% CXM and 12.2% CIP in commensal *E. coli* from children in India^38^. Most clinical and community *E. coli* isolates phenotypically resistant to CIP lacked corresponding ARGs but frequently harboured GyrA (S83L and D87N) and ParC (S80I and E84V) mutations, regardless of ARG co-occurrence. Similarly, a WGS study by Golden et al. (2021)^39^, which analysed 636 ESBL-producing *E. coli* isolates from Canadian hospitals as part of the Canadian Ward Surveillance Study (2007–2018), reported that CIP resistance was predominantly associated with QRDR mutations in GyrA (S83L and D87N) and ParC (S80I and E84V), occurring both with (n = 187) and without (n = 197) the *aac(6*′*)-Ib-cr* gene. Collectively, this reinforces the role of chromosomal QRDR mutations as the primary mechanism driving CIP resistance in *E. coli* isolates of this study. EC14 and EC19 did not carry any QRDR mutations but harboured the AcrAB-TolC efflux pump. Previous studies have shown that overexpression of this efflux pump confers resistance to CIP^40,41^. Additionally, EC19 did not harbour any ARGs conferring resistance to GEN but it carried the AcrD efflux pump, which is a member of the Resistance-Nodulation-Division (RND) family and has been reported to mediate aminoglycoside resistance^42–44^.

The prevalence of ESBL-producing *E. coli* in our study was 20% among clinical isolates and 23.5% among community isolates. However, the *bla*_CTX−M_ gene was detected by WGS in 66.7% of clinical *E. coli* isolates, yet only 20% were identified as ESBL-producers by VITEK2 AES. Similarly, clinical *K. pneumoniae*, 83.3% isolates harboured the *bla*_CTX−M−15_ but none were identified as ESBL-producers. In contrast, Carvalhaes et al. (2024)^45^ reported an AES specificity of 98.1% when detecting ESBLs in 447 clinical Enterobacterales, of which 103 were ESBL-positive by genotype and AES detected 101. This discrepancy highlights limitations of phenotypic methods and underscores the complexity of ESBL detection, particularly in the presence of co-existing resistance mechanisms^46^. Both clinical and community *E. coli* isolates exhibited resistance to 3rd generation cephalosporins (CTX/CRO and CAZ). Resistance to CTX/CRO in clinical isolates was notably high at 60%, exceeding the global median prevalence of 42% reported by the WHO (2023a)^47^, indicating a significant cephalosporin resistant *E. coli* burden in clinical settings within uMgungundlovu. The overall prevalence of CTX/CRO resistance in our *E. coli* community isolates was 23.5% (both ESBL-producing and non-ESBL-producing isolates). Shirani et al. (2019)^48^ similarly reported high CTX and CRO resistance among clinical ESBL-producing *E. coli* isolates from inpatients in Iran (73.5% and 58.8%, respectively), with lower resistance observed in non-ESBL-producers (30.3% CTX, 27.3% CRO). Similarly, Ashraf et al. (2025)^49^ reported a high prevalence of CTX and CRO resistance (80.2%) among hospital-acquired uropathogenic *E. coli* (UPEC) isolates in Pakistan, alongside a lower prevalence among community-acquired UPEC (37.9%), which is comparable to the resistance observed in our community isolates. These trends support our findings and suggest that the observed ESBL burden is consistent across different settings, reinforcing the wider dissemination of ESBL-producing strains.

In South Africa, *K. pneumoniae* exhibits high resistance rates (> 50%) to multiple antibiotic classes, with the notable exception of carbapenems^50^. In the present study, the highest resistance prevalence among clinical *K. pneumoniae* isolates was observed for CTX/CRO, CXM, CAZ, and SXT (83.3%), followed by AMC, TZP, CIP, and GEN (66.7%), and FOX (50%). Comparatively, studies conducted in KwaZulu-Natal have reported even higher resistance prevalences, with up to 100% resistance to these antibiotics among clinical *K. pneumoniae* isolates^10,13,14^. The consistently high resistance levels observed across studies likely reflect sustained antimicrobial selective pressure in hospital settings and underscore the growing challenge of managing *K. pneumoniae* infections in KwaZulu-Natal due to the substantial burden of multidrug resistance (MDR). Interestingly, clinical *K. pneumoniae* isolates did not exhibit resistance to TGC, whereas a high resistance prevalence of 50% to TGC was observed among community isolates. This finding suggests the presence of non-clinical selective pressures or potential community-associated reservoirs contributing to TGC resistance among community dwellers in uMgungundlovu. The detection of high TGC resistance in healthy community carriers is concerning, given that TGC is classified as a last-resort antibiotic for the treatment of MDR infections^51^. Similarly, Salvador-Oke et al. (2025)^52^ reported an overall 30% resistance prevalence to TGC among rectal carriage carbapenemase-producing *K. pneumoniae* isolates from community hospitals across five South African provinces. Notably, we detected both OqxA and OqxB efflux pumps in all TGC resistant isolates, irrespective of the presence of corresponding ARGs, and these efflux systems have previously been associated with reduced susceptibility to TGC^53^.

The clinical isolate KP46 harboured both *bla*_OXA−181_ and *bla*_NDM−5_ ARGs located on different contigs. The *bla*_NDM_ gene has also been reported in several South African studies^54–56^. Between 2015 and 2021, a 60% prevalence of *bla*_NDM_ genes was observed in hospitals associated with the Group for Enteric, Respiratory and Meningeal disease Surveillance in South Africa (GERMS-SA) linked to the NHLS in KwaZulu-Natal^57^. The detection of carbapenemase genes in this study therefore aligns with this broader epidemiological context and is consistent with the ongoing low-level circulation of carbapenemase-producing Enterobacterales in South Africa. All detected *bla*_OXA−181_ genes were associated with Tn*3*, IS*Kpn19*, and IS*3000*, consistent with the IS*3000*-*bla*_OXA−181_-repA-IS*Kpn19* genetic context described by Kim et al. (2021)^58^ in carbapenemase-producing *E. coli* isolates, and with findings in clinical *K. pneumoniae* reported by Madni et al. (2021)^10^ and Masalane et al. (2025)^14^, who identified Tn*3*:IS*3000*:*bla*_OXA−181_:IS*Kpn19* associations. The *bla*_NDM−5_ was associated with IS30 and IS*CR1*, aligning with the findings of Mapunda et al. (2025)^59^, who reported *bla*_NDM−5_ in IncFIB(K), ColPK3–IncX3, and IncFIB(K)–IncFII(K) plasmids flanked by a similar genetic context consisting of IS*26*, IS*30*, and IS*CR1*. We detected ColKP3 replicons in all *bla*_OXA−181_-harbouring isolates. Similarly, Takei et al. (2024)^60^ reported that all *bla*_OXA−232_ and *bla*_OXA−181_ genes were carried on ColKP3 plasmids among multidrug-resistant *K. pneumoniae* isolates recovered from two Nepalese hospitals. Notably, KP46 carried an IncX3 plasmid replicon, which has been reported to harbour *bla*_NDM−5_ in Enterobacterales, including *K. pneumoniae*, from a children’s hospital in China^61^. The IncR replicon was detected in four clinical and three community *K. pneumoniae* isolates, co-occurring with IncF replicons. IncR plasmids have been reported to carry ARGs conferring resistance to β-lactams, SXT, fluoroquinolones, aminoglycosides, tetracyclines, and chloramphenicol, and their co-residence with other plasmid replicons facilitates HGT^62,63^. These findings highlight the role of MGEs and conserved genetic contexts in the dissemination of carbapenemase genes among Enterobacterales. Collectively, these findings reinforce that the spread of carbapenemase genes is primarily driven by plasmids and conserved plasmid backbones.

The *bla*_SHV−1_ gene is typically chromosomally encoded in *K. pneumoniae*^64^ but was not detected in all isolates in this study, likely due to the emergence and diversification of other *bla*_SHV_ variants^65^. The *bla*_CTX−M−15_ ARG was predominantly detected in clinical isolates (8 *E. coli*, 5 *K. pneumoniae*), a globally dominant ESBL in *E. coli*^66^, in contrast with the higher occurrence of *bla*_TEM−1B_ among community isolates (12 *E. coli*, 2 *K. pneumoniae*), suggesting differential resistance selection between hospital and community settings. Notably, community *K. pneumoniae* isolates did not harbour any *bla*_CTX−M_ genes. Singh et al. (2023)^67^, observed a higher prevalence of the *bla*_CTX−M−15_ gene among children with recent hospitalization (hospital arm) compared to children without recent hospitalization (community arm), as well as among their family members. Their study on MDR Enterobacterales showed that *bla*_CTX−M−15_ was more predominant in the hospital arm. Consistent with our study, Belina et al. (2024)^68^ reported that *bla*_TEM−1B_ was more frequently detected than *bla*_CTX−M−15_ among diarrhoeagenic *E. coli* isolated from children under five years of age, their caretakers, and surrounding environments in Ethiopia. In *E. coli* isolates from both clinical and community settings, *bla*_CTX−M−15_ was frequently embedded within a CTX-M-15::Tn3:IS3:*qnrS1*::IS*Kpn19* genetic context and carried on plasmids. In contrast, previous studies from Pretoria and Ghana reported *bla*_CTX−M−15_ in *E. coli* to be predominantly associated with IS1380-like ISs and Tn3^8,29^. Consistent with these findings, one clinical isolate in this study (EC09) also harboured *bla*_CTX−M−15_ in association with IS*1380* (IS*Ecp1*). The *bla*_CTX−M−15_ among clinical *K. pneumoniae* isolates was associated with Tn3 and ISEcp1. Interestingly, *K. pneumoniae* ST607 clinical isolates (KP47 and KP48) carried *bla*_CTX−M−15_ flanked by Tn3 and IS*Ecp1* on both sides. Similar associations between bla_CTX−M−15_, IS*Ecp1*, and Tn3 have been reported by Masalane et al. (2025)^14^ among *K. pneumoniae* isolates from hospital patients in KwaZulu-Natal.

All gene cassettes detected were associated with *IntI1* in both settings. The *dfrA17–aadA5* cassette array within In54 was most common among clinical *E. coli* isolates, particularly ST131 isolates, whereas *dfrA14* in In191 predominated among community isolates. Similarly, Mbelle et al. (2019)^8^ reported *dfrA17–aadA5* cassette arrays in In54 among MDR *E. coli* ST131 isolates in Pretoria. The In54 has also been detected in non-ST131 *E. coli*, while In191 has been reported in clinical isolates and hospital environments in Ghana^29^. Among clinical *K. pneumoniae* isolates, the *aacA4cr–arr-3–dfrA27–aadA16* gene cassettes within In1021 was most prevalent. In contrast, only three community isolates harboured *IntI1*, two of which carried *dfrA5* and *dfrA14* gene cassettes within In13 and In191, respectively. Both In1021 and In191 have been detected in other KwaZulu-Natal clinical studies^13,14^, while *dfrA5* gene cassette has been previously reported in *K. pneumoniae* isolates from urinary tract infections in Iran^69^. These findings suggest that AMR in this study is not driven solely by clonal lineages but is also strongly shaped by horizontal gene transfer mediated by MGEs.

Community isolates of both *E. coli* and *K. pneumoniae* showed a wide diversity of STs than clinical isolates, with 23 STs detected among community isolates and 13 STs among clinical isolates. A similar observation was reported by Raffelsberger et al. (2023)^70^, who investigated ESBL-producing *E. coli* and *K. pneumoniae* carriage in a Norwegian adult population. In their study, only *E. coli* carriage isolates were compared to clinical isolates collected through the Norwegian Surveillance System for Antimicrobial Drug Resistance (NORM), revealing 58 STs among carriage isolates and 27 STs among clinical isolates. These findings suggest that clinical settings alone may underestimate the true diversity of circulating strains, highlighting the need for more genomic studies beyond the clinical context to better inform AMR surveillance in South Africa. The only common STs shared between clinical and community *E. coli* isolates were ST131, ST10, and ST1193, while for *K. pneumoniae* it was ST17. Clinical *E. coli* ST131 isolates in this study (EC03, EC09, EC10, and EC13) harboured *bla*_CTX−M−15_, consistent with findings by Louka et al. (2021)^71^, who reported that ST131 *E. coli* predominantly carried *bla*_CTX−M−15_ among hospitalized asylum seekers in the Netherlands. Notably, ST131 clinical *E. coli* isolates (EC09, EC10 and EC13) were resistant to CIP (Table 2). These findings agree with those of Ziadi et al. (2025)^72^, who reported that all clonal complex 131 (CC131) *E. coli* isolates in their study harboured *bla*_CTX−M−15_ and were resistant to CIP. Of the two ST131 in our community isolates, only EC26 was phenotypically resistant to CIP. ST1193 *E. coli* isolates have also been linked to CIP resistance in community-acquired infections across 30 county hospitals in China^73^ and in extraintestinal pathogenic *E. coli* in Vietnam^74^. Consistently, ST1193 isolates in our study (2 clinical, 1 community) were also resistant to CIP, supporting the global association of this ST with fluoroquinolone resistance. resistance. All three isolates shared the same QRDR-associated point mutations. Musicha et al. (2025)^75^ conducted a community-based longitudinal One Health cohort study in Malawi (April 2019-December 2020) and Uganda (July 2020-August 2021), including human stool samples, and reported that *E. coli* ST131 and ST10 were dominant in Malawi and Uganda, respectively. Similarly, ST10 was predominant among community *E. coli* isolates in our study. These clinical and community isolates phylogenetically clustered together, suggesting the presence of genetically related strains across both settings.

This study provides a descriptive genomic snapshot into the resistome, mobilome, and clonal diversity of *E. coli* and *K. pneumoniae* isolates from both community and clinical settings in uMgungundlovu, highlighting similarities and differences across both settings. Some of the limitations of this study were the relatively small sample size and short sampling period which does not fully capture the resistome and mobilome diversity and dynamics of AMR in this region. Additionally, ESBL detection in clinical isolates was based on the VITEK 2 system without confirmatory testing, which may have contributed to an underestimation of ESBL-producing isolates. The observed similarities between isolates from both settings may suggest the presence of shared selective pressures; however, further longitudinal and epidemiological investigations are required to confirm these relationships.

## Conclusion

Overall, clinical isolates demonstrated a trend toward more extensive and diverse antibiograms than community isolates for both *E. coli* and *K. pneumoniae*; however, these differences were largely descriptive, with most phenotypic resistance patterns and resistome profiles not showing statistically significant differences between the two settings. Diverse resistomes, mobilomes, and clonalities were identified in both settings, with several overlapping across clinical and community isolates. Although resistance levels were generally higher in clinical isolates, the presence of MDR genes and MGEs in community isolates underscores the potential of healthy carriers as silent reservoirs. Notably, clinical isolates were collected from varied specimen types, while community isolates were limited to stool samples. This difference in source context may affect resistome and mobilome profiles and limits direct comparability. Phylogenetic clustering demonstrated genetic relatedness between isolates from both settings, suggesting overlap of shared lineages across community and clinical settings. These findings reinforce that community settings should not be overlooked in AMR surveillance and mitigation efforts.

## Supplementary Information

Below is the link to the electronic supplementary material.

Supplementary Material 1

Supplementary Material 2

## Data Availability

All analysed data are included in the manuscript and its supplementary materials. The assembled genomes of this study are deposited in the NCBI GenBank database under the BioProject PRJNA1183563.
