# Supplementary material for: Community and clinical resistomes and mobilomes: a comparative genomic snapshot of Escherichia coli and Klebsiella pneumoniae
Source: Sci Rep. 2026 Jul 28;16:34488. doi: 10.1038/s41598-026-64511-x (PMC13710198; doi:10.1038/s41598-026-64511-x)
Supplement: Supplementary file 1 [file 41598_2026_64511_MOESM1_ESM.docx]

**Table S1:** Participant demographics and genomic characteristics of sequenced *E. coli* and *K. pneumoniae* isolates from clinical and community samples

| **Isolate ID** | **Accession no.** | **Species** | **Sex** | **Age (years)** | **Specimen type** | **Setting** | **Sequence size (bp)** | **No. of contigs** | **GC content (%)** | **N50** | **L50** |
| --- | --- | --- | --- | --- | --- | --- | --- | --- | --- | --- | --- |
| EC01 | SAMN44628981 | *E. coli* | F | 55 | Urine | Clinical | 4664742 | 192 | 50.7 | 74038 | 20 |
| EC02 | SAMN44628982 | *E. coli* | F | 81 | Urine | Clinical | 5470388 | 688 | 50.6 | 39368 | 45 |
| EC03 | SAMN44628983 | *E. coli* | F | <1 year | Urine | Clinical | 5480363 | 368 | 51.9 | 208575 | 9 |
| EC04 | SAMN44628984 | *E. coli* | F | 26 | Tissue | Clinical | 5215906 | 91 | 50.6 | 155771 | 10 |
| EC05 | SAMN44628985 | *E. coli* | F | 60 | Urine | Clinical | 5273316 | 104 | 50.6 | 120591 | 16 |
| EC07 | SAMN44628987 | *E. coli* | M | 92 | Urine | Clinical | 5744267 | 1468 | 50.7 | 17374 | 94 |
| EC08 | SAMN44628988 | *E. coli* | F | 21 | Urine | Clinical | 5239615 | 136 | 50.7 | 114193 | 14 |
| EC09 | SAMN44628989 | *E. coli* | F | 75 | Urine | Clinical | 5257170 | 346 | 50.7 | 77633 | 21 |
| EC10 | SAMN44628990 | *E. coli* | F | 33 | Tissue | Clinical | 4985625 | 82 | 50.8 | 225684 | 7 |
| EC13 | SAMN44628993 | *E. coli* | M | 41 | Blood culture | Clinical | 5255080 | 103 | 50.8 | 158933 | 10 |
| EC14 | SAMN44628994 | *E. coli* | M | 38 | Blood culture | Clinical | 4822768 | 129 | 50.8 | 95797 | 18 |
| EC15 | SAMN44628995 | *E. coli* | F | 17 | Urine | Clinical | 4932441 | 134 | 50.7 | 121143 | 12 |
| EC16 | SAMN44628996 | *E. coli* | M | 42 | Urine | Clinical | 5164990 | 311 | 50.6 | 92529 | 20 |
| EC18 | SAMN44628998 | *E. coli* | F | 56 | Catheter tip | Clinical | 4899046 | 86 | 50.6 | 121143 | 13 |
| EC19 | SAMN44628999 | *E. coli* | M | 76 | Fluid-aspirate | Clinical | 4721617 | 100 | 50.7 | 138046 | 10 |
| EC21 | SAMN44629001 | *E. coli* | F | 36 | Stool | Community | 4947919 | 90 | 50.6 | 258770 | 6 |
| EC22 | SAMN44629002 | *E. coli* | F | 62 | Stool | Community | 5118429 | 103 | 50.6 | 152654 | 11 |
| EC23 | SAMN44629003 | *E. coli* | F | 65 | Stool | Community | 5170098 | 141 | 50.4 | 110327 | 18 |
| EC24 | SAMN44629004 | *E. coli* | M | 27 | Stool | Community | 6179188 | 1251 | 50.5 | 23059 | 70 |
| EC25 | SAMN44629005 | *E. coli* | F | 50 | Stool | Community | 5163936 | 130 | 50.4 | 118612 | 16 |
| EC26 | SAMN44629006 | *E. coli* | F | 37 | Stool | Community | 5067166 | 87 | 50.7 | 159739 | 12 |
| EC30 | SAMN44629010 | *E. coli* | F | 55 | Stool | Community | 4809910 | 225 | 50.5 | 67628 | 22 |
| EC31 | SAMN44629011 | *E. coli* | F | 54 | Stool | Community | 4688321 | 59 | 50.8 | 182215 | 10 |
| EC32 | SAMN44629012 | *E. coli* | M | 31 | Stool | Community | 5080504 | 173 | 50.6 | 84590 | 20 |
| EC33 | SAMN44629013 | *E. coli* | F | 37 | Stool | Community | 5839137 | 2392 | 50.4 | 3859 | 402 |
| EC35 | SAMN44629015 | *E. coli* | F | 38 | Stool | Community | 4996882 | 92 | 50.5 | 215054 | 8 |
| EC36 | SAMN44629016 | *E. coli* | N/A | N/A | Stool | Community | 5008901 | 64 | 50.6 | 235774 | 8 |
| EC37 | SAMN44629017 | *E. coli* | N/A | N/A | Stool | Community | 5161183 | 66 | 50.7 | 195158 | 9 |
| EC38 | SAMN44629018 | *E. coli* | F | 39 | Stool | Community | 5135473 | 116 | 50.7 | 169840 | 12 |
| EC39 | SAMN44629019 | *E. coli* | M | 28 | Stool | Community | 5089382 | 142 | 50.7 | 126316 | 13 |
| EC40 | SAMN44629020 | *E. coli* | F | 31 | Stool | Community | 4916063 | 137 | 50.7 | 127336 | 12 |
| EC41 | SAMN44629021 | *E. coli* | F | 26 | Stool | Community | 5137122 | 143 | 50.7 | 140993 | 12 |
| KP43 | SAMN44629023 | *K. pneumoniae* | M | 20 | Urine | Clinical | 5697749 | 203 | 57.0 | 198204 | 10 |
| KP45 | SAMN44629025 | *K. pneumoniae* | F | 36 | Urine | Clinical | 5691941 | 208 | 57.0 | 156059 | 12 |
| KP46 | SAMN44629026 | *K. pneumoniae* | F | 50 | Urine | Clinical | 5725555 | 121 | 57.0 | 263913 | 7 |
| KP47 | SAMN44629027 | *K. pneumoniae* | M | <1 year | Blood culture | Clinical | 5556217 | 105 | 57.2 | 217065 | 9 |
| KP48 | SAMN44629028 | *K. pneumoniae* | M | <1 year | Blood culture | Clinical | 5678080 | 219 | 57.1 | 172178 | 10 |
| KP51 | SAMN44629031 | *K. pneumoniae* | F | <1 year | Blood culture | Clinical | 5645123 | 230 | 57.1 | 161736 | 12 |
| EC27 | SAMN44629007 | *K. pneumoniae* | F | 63 | Stool | Community | 5525333 | 69 | 57.0 | 367077 | 6 |
| KP53 | SAMN44629033 | *K. pneumoniae* | M | 27 | Stool | Community | 5563757 | 52 | 56.9 | 277243 | 7 |
| KP54 | SAMN44629034 | *K. pneumoniae* | F | 52 | Stool | Community | 5181281 | 34 | 57.6 | 397451 | 6 |
| KP55 | SAMN44629035 | *K. pneumoniae* | F | 63 | Stool | Community | 5502431 | 124 | 57.2 | 197398 | 11 |
| KP56 | SAMN44629036 | *K. pneumoniae* | M | 35 | Stool | Community | 5359929 | 95 | 57.3 | 142403 | 11 |
| KP57 | SAMN44629037 | *K. pneumoniae* | F | 35 | Stool | Community | 6387976 | 515 | 56.9 | 46189 | 37 |
| KP58 | SAMN44629038 | *K. pneumoniae* | F | 27 | Stool | Community | 5683501 | 74 | 57.1 | 208034 | 10 |
| KP59 | SAMN44629039 | *K. pneumoniae* | F | 22 | Stool | Community | 5284675 | 32 | 57.4 | 443289 | 5 |

**Key:** N50 = smallest contig of the size-sorted contigs that make up at least 50% of the respective assembly; L50 = number of contigs that make up at least 50% of the respective total assembly length

**Table S2:** Fluoroquinolone-associated point mutations detected in CIP resistant *E. coli* isolates

| **Isolate ID** | **Setting** | **Fluoroquinolone point mutations** | | | |
| --- | --- | --- | --- | --- | --- |
|  |  | **GyrA** | **GyrB** | **ParC** | **ParE** |
| EC01^Δ^ | Clinical | S83L, D87N | E219K* | S80I, A56T, A620V* | - |
| EC02^Δ^ | Clinical | S83L, D87N, D678E*, A828S* | A618T* | S80I | L416F, L254Q* |
| EC04^Δ^ | Clinical | S83L, D678E* | - | D475E*, Q695L* | - |
| EC07^Δ^ | Clinical | - | A618T* | - | - |
| EC09^Δ^ | Clinical | S83L, D87N, A828S* | A618T* | S80I, E84V, A192V*, A471G*, D475E*, Q481H* | I529L, V136I* |
| EC10^Δ^ | Clinical | S83L, A828S* | A618T* | S80I, E84V, A192V*, A471G*, D475E*, Q481H* | I529L, V136I* |
| EC13^#^ | Clinical | S83L, D87N, A828S* | A618T* | S80I, E84V, A192V*, A471G*, D475E*, Q481H* | I529L, V136I* |
| EC14^#^ | Clinical |  | - | - | - |
| EC16^#^ | Clinical | S83L, D87N, D678E*, A828S* | A618T* | S80I | L416F, L254Q* |
| EC18^#^ | Clinical | N652H* | A155V*, D639N* | - | - |
| EC19^Δ^ | Clinical | - | - | - | - |
| EC22^Δ^ | Community | S83L, D87N, D678E*, A828S* | A618T* | S80I | L416F, L254Q* |
| EC26^Δ^ | Community | S83L, D87N, A828S* | A618T* | S80I, E84V, A192V*, A471G*, D475E*, Q481H* | I529L, V136I* |

^Δ^CIP resistant isolates without corresponding ARGs; ^#^CIP resistant isolates with corresponding ARGs; *Novel mutations

**Table S3:** Genomic distribution of MGEs and their association with ARGs in clinical and community *E. coli* isolates

| **Isolate (MLST)** | **Setting** | **Contig** | **Synteny of ARGs with MGEs** | **Plasmid/chromosomal sequence with closest nucleotide homology (accession number)** |
| --- | --- | --- | --- | --- |
| EC01 (ST3337) | Clinical | 76 | *CatA1::Tn3 (TnAs3):::IntI1:DfrA17:AadA5:QacE∆1:sul1:::IS6100* | *E. coli strain* 800 chromosome (CP157958.1) |
|  |  | 100 | *tet(B):::IS4 (ISVsa5)* | *E. coli* strain OXEC-466 plasmid unnamed (CP165369.1) |
|  |  | 118 | *Transposase:TEM-1* | *E. coli* strain OXEC-478 plasmid unnamed (CP165316.1) |
| EC02 (ST1193) | Clinical | 123 | *IntI1:dfrA17:AadA5:QacE∆1:sul1:::IS6 (IS6100):::Mph(A)* | *E. coli* 09-02E plasmid p1-09-02E DNA (AP022651.1) |
|  |  | 227 | *Transposase:CTX-M-27:IS5 family transposase* | *E. coli* 09-02E plasmid p1-09-02E DNA (AP022651.1) |
| EC03 (ST131) | Clinical | 52 | *IntI1:DfrA17:AadA5:QacE∆1:sul1::::IS6 (IS6100):::Mph(A)* | *E. coli* strain S1-IND-01-B plasmid p1-S1-IND-01-B (CP145656.1) |
|  |  | 74 | *ermB::transposase* | *E. coli* strain S1-IVC-02-A plasmid p1-S1-IVC-02-A (CP145699.1) |
| EC04 (ST38) | Clinical | 61 | *IS6100:::Mph(A)* | *E. coli* strain W46 isolate China: Kunming chromosome (CP163115.1) |
|  |  | 66 | *Transposase:CTX-M-15* | *E. coli* strain E305-2 plasmid punnamed2 (CP090252.1) |
|  |  | 62 | *sul2:IS91 (ISVsa3 transposase)* | *E. coli* strain SCU-164 chromosome (CP054343.1) |
|  |  | 65 | *IS3 family transposase::Aac(3)-IIe* | *E. coli* strain SX4 plasmid pSX4-2-ESBLs (CP142399.1) |
| EC05 (ST38) | Clinical | 37 | *IntI1:DfrA1:AadA1:QacE∆1:sul1:::transposase:::::::::TEM-1::Tn3:::tet(A):::TnAs1:IS3(IS1133):Aph(3'')-Ib:Aph(6)-Id* | *E. coli* strain OXEC-285 plasmid p2 (CP164926.1) |
| EC07 (ST3572) | Clinical | 9 | *Tn3 transposase:IS3000 transposase:OXA-181:::ISKra4(ISKpn19 transposase)* | *E. coli* strain Eco-15 plasmid pEco15-4 (CP047714.1) |
|  |  | 182 | *QacE∆1:sul1:IS110(IS4321)::IS6(IS6100):::Mph(A)* | *E. coli* strain 15.TR.026_OXA chromosome (CP032145.1) |
|  |  | 369 | *DfrA17:IntI1* | *E. coli* strain SX23 plasmid pSX23-2-ESBLs (CP142790.1) |
| EC08 (ST69) | Clinical | 92 | *AadA3:DfrA17:IntI1* | *E. coli* strain SCU-482 plasmid pSCU-482-1 (CP053248.1) |
|  |  | 85 | *DDE-type integrase:::Mph(A)* | *E. coli* strain GN4143 plasmid pGN4143-1 (CP142071.1) |
|  |  | 74 | *tet(B)::IS4(ISVsa5)* | *E. coli* strain OXEC-466 plasmid unnamed (CP165369.1) |
| EC09 (ST131) | Clinical | 93 | *IntI1:DfrA17:AadA5:QacE∆1:sul1:::IS6 (IS6100):::Mph(A):IS6 family transposase* | *E. coli* strain KFS-D30 plasmid pKFS-D30_1 (CP125005.1) |
|  |  | 153 | *IS4(ISVsa5 transposase):Aac(3)-IId* | *E. coli* strain WTP01 plasmid pWTP-01 (OR287786.1) |
|  |  | 99 | *IS1380 (ISEcp1 transposase):CTX-M-15::Tn3 family transposase* | *E. coli* strain 98214 chromosome (CP173519.1) |
|  |  | 104 | *IS1 transposase: TEM-1* | *E. coli* strain EcPF18 plasmid p1 (CP054220.1) |
| EC10 (ST131) | Clinical | 52 | *IntI1:DfrA17:AadA5:QacE∆1:sul1:::IS6(IS6100 transposase):::Mph(A)* | *E. coli* strain S1-IND-01-B plasmid p1-S1-IND-01-B (CP145656.1) |
|  |  | 61 | *ermB:transposase* | *E. coli* strain S1-IVC-02-A plasmid p1-S1-IVC-02-A (CP145699.1) |
| EC13 (ST131) | Clinical | 45 | *IntI1:DfrA17:AadA5:QacE∆1:sul1:::IS6(IS6100 transposase):::Mph(A)* | *E. coli* O25b:H4-ST131 strain 418 plasmid p418 (MK295833.1) |
|  |  | 61 | *CTX-M-15::Tn3 transposase* | *E. coli* strain IVRI_FBI_607 chromosome (CP125922.1) |
|  |  | 57 | *tet(A):::Tn3 transposase* | *E. coli* strain GN4143 plasmid pGN4143-1 (CP142071.1) |
| EC14 (ST10) | Clinical | 68 | *IntI1: DfrA17:AadA5:QacE∆1:sul1:::IS6(IS6100 transposase):::Mph(A)* | *E. coli* strain 18AR0845 plasmid P18ar0845a (CP175692.1) |
|  |  | 60 | *IS1 transposase:IS6(IS26 transposase):CTX-M-15::Tn3 transposase:IS3 transposase: QnrS1::ISKra4(ISKpn19 transposase)* | *E. coli* strain C51 plasmid pC5103 (CP127312.1) |
| EC15 (ST13823) | Clinical | 57 | *CTX-M-15::Tn3 transposase:IS3 transposase:QnrS1::ISKra4 (ISKpn19 transposase):::sul1:QacE∆1:AadA5:DfrA17:IntI1* | *E. coli* strain 129130 plasmid pMRSN129130_p3 (CP158336.1) |
| EC16 (ST1193) | Clinical | 62 | *Mph(A):::IS6(IS6100 transposase)::TEM-1:IS1 transposase* | *E. coli* isolate 30345_1#221 genome assembly, plasmid: 2 (OZ039844.1) |
|  |  | 79 | *ISKra4(ISKpn19 transposase)::QnrS1:IS3 transposase:Tn3 transposase::CTX-M-231:IS6 (IS26 family transposase)* | *E. coli* strain 123636 plasmid pMRSN123636_p3 (CP158326.1) |
|  |  | 100 | *ermB:::transposase* | *E. coli* strain 5M plasmid pISV_IncFII_NDM-5 (MN218686.1) |
| EC18 (ST13823) | Clinical | 57 | *CTX-M-15::Tn3 transposase:IS3 transposase:QnrS1::ISKra4 (ISKpn19 transposase):::sul1:QacE∆1:AadA5:DfrA17:IntI1* | *E. coli* strain 129130 plasmid pMRSN129130_p3 (CP158336.1) |
| EC19 (ST3572) | Clinical | 29 | *IS6(IS26 transposase):Tn3 transposase:Tn3(IS3000 transposase):OXA-181:::ISKra4(ISKpn19 transposase)* | *E. coli* strain Eco-15 plasmid pEco15-4 (CP047714.1) |
|  |  | 51 | *Tn3(TnAs1 transposase):::IntI1:DfrA17:QacE∆1:sul1:IS110(IS4321 transposase):::IS6(IS6100 transposase):::Mph(A)* | *E. coli* strain 15.TR.026_OXA chromosome (CP032145.1) |
| EC21 (ST1722) | Community | 49 | *QacE∆1:AadA1: DfrA1:IntI1* | *E. coli* strain JKHS016 plasmid pJKHS016_2 (CP147060.1) |
|  |  | 32 | *ISKra4 transposase::QnrS1:IS3 transposase:Tn3 transposase::CTX-M-15* | *E. coli* plasmid pWP8-S18-ESBL-07_2 DNA (AP022263.1) |
|  |  | 43 | *TEM-216: TnAs1 transposase:IS91 transposase:sul2* | *E. coli* strain 786605 plasmid p786605_1 (CP091395.1) |
| EC22 (ST1193) | Community | 67 | *DDE-type integrase:::Mph(A)* | *E. coli* strain GN4143 plasmid pGN4143-1 (CP142071.1) |
|  |  | 59 | *tet(B)::IS4(ISVsa5 transposase):IS1 transposase* | *E. coli* strain OXEC-466 plasmid unnamed (CP165369.1) |
|  |  | 71 | *IS1 transposase:DfrA17:IntI1* | *E. coli* strain EcPF40 plasmid p1 (CP054215.1) |
| EC23 (ST10) | Community | 71 | *TEM-1:Aph(6)-Id:Aph(3'')-Ib:sul2::IS110(IS5075 transposase):Tn3 transposase* | *E. coli* strain EC28 plasmid p2 (CP049102.1) |
|  |  | 86 | *Tn3 transposase:::tet(A)* | *E. coli* strain IVRI_FBI_650 chromosome (CP125923.1) |
|  |  | 103 | *DfrA14:IntI1* | *E. coli* strain AR Bank #0349 plasmid pAR349 (CP041997.1) |
| EC24 (ST1380) | Community | 5 | *sul2::DfrA14:Aph(3'')-Ib:Aph(6)-Id:IS91 transposase* | *E. coli* strain 31CH-1 plasmid p31CH-1 (PP566049.1) |
|  |  | 235 | *Tn3 transposase::tet(A):::Tn3 transposase* | *E. coli* strain IVRI_FBI_650 chromosome (CP125923.1) |
| EC25 (ST10) | Community | 69 | *TEM-1:Aph(6)-Id:Aph(3'')-Ib:sul2::IS110(IS5075 transposase):Tn3 transposase* | *E. coli* strain EC28 plasmid p2 (CP049102.1) |
|  |  | 76 | *Tn3(TnAs1 transposase):::tet(A)* | *E. coli* strain IVRI_FBI_650 chromosome (CP125923.1) |
|  |  | 90 | *DfrA14:IntI1* | *E. coli* strain AR Bank #0349 plasmid pAR349 (CP041997.1) |
| EC26 (ST131) | Community | 71 | *CTX-M-27:IS5/IS1182 transposase* | *E. coli* O25b:H4-ST131 strain 4224 plasmid pMB6910_1 (CP174354.1) |
| EC30 (ST2758) | Community | 82 | *IS3 transposase:QnrS1::ISKra4 (ISKpn19 transposase):::IS91 transposase:Aph(6)-Id:Aph(3'')-Ib:sul2::IS110 (IS5075 transposase):Tn3 transposase* | *E. coli* strain 1008S19 plasmid p1008S19_1 (CP147051.1) |
|  |  | 118 | *DfrA14:IntI1* | *E. col*i strain CFSAN061766 plasmid pCFSAN061766 (CP042872.1) |
| EC31 (ST2852) | Community | 40 | *Tn3 transposase:::ISKra4 (ISKpn19 transposase)::QnrS1:IS3 transposase:Tn3 transposase::TEM-1:Tn3 transposase:::tet(A)* | *E. coli* strain KFu021 plasmid pKFu021_1 (CP147102.1) |
|  |  | 43 | *Tn3 transposase:IS110(IS5075 transposase)::sul2:IS91(ISVsa3 transposase)* | *E. coli* strain KFu021 plasmid pKFu021_1 (CP147102.1) |
|  |  | 49 | *DfrA14:IntI1* | *E. coli* strain 98224 plasmid p98224-contig_3 (CP173471.1) |
| EC32 (ST382) | Community | 98 | *Aph(6)-Id:Aph(3'')-Ib:sul2:::IS6(IS26 transposase)* | *E. coli* strain OXEC-448 chromosome (CP165428.1) |
|  |  | 86 | *TEM-1:::QacE∆1:DfrA7:IntI1:::TnAs3 transposase* | *E. coli* O104:H4 strain FWSEC0009 chromosome (CP031902.1) |
|  |  | 95 | *tet(B):::IS4(ISVsa5 transposase):IS1 transposase* | *E. coli* strain OXEC-466 plasmid unnamed (CP165369.1) |
| EC33 (ST336) | Community | 19 | *Tn3 transposase:::ISKra4 (ISKpn19 transposase)::QnrS1:IS3 transposase:Tn3 transposase::TEM-1:Tn3 transposase:::tet(A):::Tn3 transposase* | *E. coli* strain KFu021 plasmid pKFu021_1 (CP147102.1) |
|  |  | 251 | *sul2:IS91(ISVsa3 transposase)* | *E. coli* strain KKa014 plasmid pKKa014_1 (CP147106.1) |
|  |  | 991 | *DfrA14:IntI1* | *E. coli* strain 98224 plasmid p98224-contig_3 (CP173471.1) |
| EC35 (ST2178) | Community | 56 | *QacE∆1:DfrA7:IntI1:::TnAs3 transposase* | *E. coli* strain SCU-164 chromosome (CP054343.1) |
| EC36 (ST101) | Community | 34 | *sul1:QacE∆1:AadA1:DfrA1:IntI1:::IS1 transposase* | *E. coli* strain HB37 chromosome HB37 (CP053080.1) |
|  |  | 39 | *IS5* *transposase::IS5(IS903B transposase):Aph(6)-Id* | *E. coli* strain 58-3 plasmid pCD58-3-1 (CP050037.1) |
|  |  | 42 | *TnAs1 transposase:::tet(A)* | *E. coli* strain WWCOL-236 plasmid pCOL236-1 (CP178553.1) |
| EC37 (ST131) | Community | 39 | *sul**1:QacE∆1:AadA5:DfrA17:IntI1* | *E. coli* strain SCU-482 plasmid pSCU-482-1 (CP053248.1) |
|  |  | 45 | *DDE-type integrase:::Mph(A)* | *E. coli* strain GN4143 plasmid pGN4143-1 (CP142071.1) |
| EC38 (ST4406) | Community | 2 | *IS1(IS1B transposase):CatA1::TnAs3 transposase:::IntI1:DfrA7:QacE∆1* | *E. coli* strain RHB23-C03 chromosome (CP057559.1) |
| EC39 (ST3570) | Community | 51 | *Tn3 transposase::TEM-1:IS1 transposase* | *E. coli* strain C012_chr chromosome (CP119577.1) |
|  |  | 53 | *IS21(IS1326 transposase):IS21(IS1326 transposase)::sul1:QacE∆1:DfrA5:IntI1:::TnAs3 transposase::CatA1:IS1 transposase* | *E. coli* strain PNUSAE013304 chromosome (CP034936.1) |
| EC40 (ST13823) | Community | 56 | *CTX-M-15::Tn3 transposase:IS3 transposase:QnrS1::ISKra4 (ISKpn19 transposase):::sul1:QacE∆1:AadA5:DfrA17:IntI1* | *E. coli* strain 129130 plasmid pMRSN129130_p3 (CP158336.1) |
| EC41 (ST210) | Community | 48 | *Mph(A):::IS6(IS6100 transposase)::TEM-1:IS1 transposase* | *E. coli* isolate 30348_1#299 genome assembly, plasmid: 2 (OZ038863.1) |
|  |  | 55 | *ISKra4(ISKpn19 transposase)::QnrS1:IS3 transposase:Tn3 transposase::CTX-M-231:IS6 (IS26 family transposase)* | *E. coli* strain 123636 plasmid pMRSN123636_p3 (CP158326.1) |
|  |  | 70 | *ermB:::transposase* | *E. coli* strain 5M plasmid pISV_IncFII_NDM-5 (MN218686.1) |

**Table S4:** Genomic distribution of MGEs and their association with ARGs in clinical and community *K. pneumoniae* isolates

| **Isolate ID (MLST)** | **Setting** | **Contig** | **Synteny of ARGs with MGEs** | **Plasmid/chromosomal sequence with closest nucleotide homology (accession number)** |
| --- | --- | --- | --- | --- |
| KP45 (ST348) | Clinical | 47 | *IS110(IS5075 transposase)::sul2:Aph(3'')-Ib:Aph(6)-Id:IS91 transposase* | *K. pneumoniae* subsp. *pneumoniae* strain ST101:960186733 plasmid p19-10_01 (CP023488.1) |
|  |  | 1 | *IS3 transposase:integrase::::::OqxA:OqxB25:::::::::::: emrA:emrB* | *K. pneumoniae* strain ARLG-4837 chromosome (CP067750.1) |
|  |  | 57 | *IS1 transposase::::IS6(IS6100 transposase):::DfrA14:IntI1* | *K. pneumoniae* strain AR_0109 plasmid unnamed6 (CP032212.1) |
| KP46 (ST17) | Clinical | 46 | *Aac(3)-IIe:IS3 transposase::IS3(ISKpn11 transposase)::IS5(ISKpn12 transposase)* | *K. pneumoniae* strain Kp202 plasmid pKp202_2 (CP041084.1) |
|  |  | 61 | *AadA16:DfrA27:Arr-3:Aac(6')-Ib-cr5:IntI1* | *K. pneumoniae* strain KP09 plasmid pKP09-CTX-M (CP178566.1) |
|  |  | 42 | *Tn3 transposase:IS3000 transposase:OXA-181:::ISKra4(ISKpn19 transposase):::::QnrS1:transposase* | *K. pneumoniae* strain 709 plasmid pKP709-OXA181 (MN227183.1) |
|  |  | 52 | *IS30 transposase:NDM-5::::IS91(ISCR1 transposase)* | *K. pneumoniae* strain 47733 plasmid p47733_NDM_5 (CP050367.1) |
|  |  | 66 | *IS1380(ISEcp1 transposase):CTX-M-15* | *K. pneumoniae* strain KP09 plasmid pKP09-CTX-M (CP178566.1) |
|  |  | 4 | *IS3 transposase:::::::OqxA:OqxB25:::::::::::::emrA:emrB* | *K. pneumoniae* strain NK_H8_118 chromosome (CP152537.1) |
| KP47 (ST607) | Clinical | 45 | *AadA16:DfrA27:Arr-3:Aac(6')-Ib-cr5:IntI1:* | *K. pneumoniae* strain Bckp021 plasmid pBckp021-1 (CP050835.1) |
|  |  | 48 | *IS5(ISKpn12 transposase)::IS3(ISKpn11 transposase)::IS3 transposase:Aac(3)-IIe:TEM-1* | *K. pneumoniae* strain MAKM-3381 chromosome (CP129122.1) |
|  |  | 54 | *IS1380(ISEcp1 transposase):CTX-M-15::Tn3 transposase* | *K. pneumoniae* strain GN4146 plasmid pGN4146-1 (CP142061.1) |
|  |  | 56 | *QnrB6::IS91(ISCR1 transposase)* | *K. pneumoniae* strain E16KP0115 plasmid unnamed (CP052638.1) |
| KP48 (ST607) | Clinical | 46 | *IS5(ISKpn12 transposase)::IS3(ISKpn11 transposase)::IS3 transposase:Aac(3)-IIe:TEM-1:::IS1380(ISEcp1 transposase): CTX-M-15::Tn3 transposase* | *K. pneumoniae* strain MAKM-3381 chromosome (CP129122.1) |
|  |  | 49 | *AadA16:DfrA27:Arr-3:Aac(6')-Ib-cr5:IntI1* | *K. pneumoniae* strain Bckp021 plasmid pBckp021-1 (CP050835.1) |
|  |  | 71 | *Aph(6)-Id:IS91 transposase* | *K. pneumoniae* strain C16KP0160 plasmid pC16KP0160-4 (CP052740.1) |
|  |  | 63 | *QnrB6::IS91(ISCR1 transposase)* | *K. pneumoniae* strain E16KP0115 plasmid unnamed (CP052638.1) |
| KP51 (ST133) | Clinical | 52 | *AadA16:DfrA27:Arr-3:Aac(6')-Ib-cr5:IntI1* | *K. pneumoniae* strain Bckp021 plasmid pBckp021-1 (CP050835.1) |
|  |  | 54 | *ISPa38 transposase:::::Aph(6)-Id:Aph(3'')-Ib:sul2* | *K. pneumoniae* strain E128606 plasmid pE128606.6 (CP136391.1) |
|  |  | 61 | *IS3(ISKpn11 transposase):Aac(3)-IIe:TEM-34:::IS1380(ISEcp1 transposase)* | *K. pneumoniae* strain MAKM-3381 chromosome (CP129122.1) |
|  |  | 21 | *emrB:emrA:::::::::::::OqxB14:OqxA:::::integrase:IS3 transposase* | *K. pneumoniae* strain NK_H15_032 chromosome (CP152854.1) |
|  |  | 69 | *QnrB6::IS91(ISCR1 transposase)* | *K. pneumoniae* strain E16KP0115 plasmid unnamed (CP052638.1) |
| EC27 | Community | 50 | *sul1:QacE∆1:DfrA5:IntI1* | *K. pneumoniae* strain KP06_UID2108280006 plasmid unnamed1 (CP171581.1) |
| KP53 (ST193) | Community | 33 | *mef(B):::sul3:IS256 transposase:qacL:AadA1:CmlA1:::IntI1:::TnAs1 transposase* | *K. pneumoniae* strain SRY516 plasmid unnamed1 (CP138715.1) |
|  |  | 24 | *ISKra4(ISKpn19 transposase)::QnrS1:IS3 transposase:LAP-2* | *K. pneumoniae* subsp. pneumoniae strain SCKP020079 plasmid pLAP2_020079 (CP029382.1) |
|  |  | 36 | *Tn3 transposase:IS110(IS5075 transposase)::sul2:IS91(ISVsa3 transposase)* | *K. pneumoniae* strain KP20194c4 plasmid pKP20194c4-p3 (CP054747.1) |
|  |  | 38 | *TnAs1 transposase:::tet(A)* | *K. pneumoniae* strain GN4147 plasmid pGN4147-2 (CP142055.1) |
| KP55 (ST17) | Community | 2 | *IS3 transposase:::::: OqxA:OqxB25::::::::::::: emrA:emrB* | *K. pneumoniae* strain SB1067 chromosome (CP084860.1) (99 %) |
| KP57 (ST45) | Community | 169 | *Mph(A):::IS6(IS6100 transposase):::DfrA14:IntI1* | *K. pneumoniae* subsp. pneumoniae strain R210-2 plasmid pR210-2-VIM (CP034084.1) |
| KP58 (ST873) | Community | 50 | *sul2:IS91(ISVsa3 transposase)* | *K. pneumoniae* strain 1675474 plasmid p1675474_1 (MK649827.1) |
| KP59 (ST1863) | Community | 21 | *TEM-1::IS6 transposase::::IS6(IS26 transposase):Tn3 transposase:IS3 transposase:QnrS1:ISKra4(ISKpn19 transposase)* | *K. pneumoniae* strain S166-1 plasmid pS166-1.4 (CP063949.1) |
